# Recent Progress of Photothermal Therapy Based on Conjugated Nanomaterials in Combating Microbial Infections

**DOI:** 10.3390/nano13152269

**Published:** 2023-08-07

**Authors:** Yue Zhao, Yi Wang, Xiaoyu Wang, Ruilian Qi, Huanxiang Yuan

**Affiliations:** 1 Department of Chemistry, College of Chemistry and Materials Engineering, Beijing Technology and Business University, Beijing 100048, China; 2 School of Materials Science and Engineering, University of Science and Technology Beijing, Beijing 100083, China

**Keywords:** photothermal therapy, conjugated nanomaterials, antimicrobial activity

## Abstract

Photothermal therapy has the advantages of non-invasiveness, low toxicity, simple operation, a broad spectrum of antibacterial ability, and non-proneness to developing drug resistance, which provide it with irreplaceable superiority in fighting against microbial infection. The effect of photothermal therapy is closely related to the choice of photothermal agent. Conjugated nanomaterials are potential candidates for photothermal agents because of their easy modification, excellent photothermal conversion efficiency, good photostability, and biodegradability. In this paper, the application of photothermal agents based on conjugated nanomaterials in photothermal antimicrobial treatment is reviewed, including conjugated small molecules, conjugated oligomers, conjugated polymers, and pseudo-conjugated polymers. At the same time, the application of conjugated nanomaterials in the combination of photothermal therapy (PTT) and photodynamic therapy (PDT) is briefly introduced. Finally, the research status, limitations, and prospects of photothermal therapy using conjugated nanomaterials as photothermal agents are discussed.

## 1. Introduction

Microbial infection is a global public health problem, and it can cause many diseases that are difficult to cure, such as tuberculosis and infectious diarrhea [1]. In the 20th century, the discovery of antibiotics symbolized the great progress of modern medicine, as antibiotics could effectively treat diseases caused by microbial infections by inhibiting the synthesis of bacterial cell walls and destroying the structure of cell membranes, saving millions of lives [2,3]. However, the abuse of antibiotics has led to the gradual emergence of bacteria with multi-drug resistance (MDR), which can no longer be treated with traditional treatment methods and have resulted in more serious medical problems [4]. Among the many MDR strains, ESKAPE pathogens (*Enterococcus* sp., *Staphylococcus aureus*, *Klebsiella pneumoniae*, *Acinetobacter baumannii*, *Pseudomonas aeruginosa,* and *Enterobacter* spp.) cause higher infection rates and mortality, which have attracted great attention in related research and clinical treatment [5,6]. Currently, 700,000 people die each year because of MDR [7], and statistics predict that 10 million people worldwide could die due to superbugs by 2025 [8]. One of the traditional solutions is the combined use of two or more kinds of antibiotics, which can achieve better antibacterial effects and reduce side effects while giving a small dose [9]. For example, the combination of trimethoprim (TMP) and sulfamethoxazole (SMX) is a common synergistic antibacterial combination. TMP can inhibit the production of dihydrotrexate pyrophosphate (DHPPP), which is a precursor of folate, thereby enhancing the inhibitory effect of SMX on tetrahydrofolate (THF) and realizing synergistic treatment of microbial infections [10,11]. However, this method still has the risk of causing MDR and does not solve the issue fundamentally.

With the development and progress of science and technology, more and more therapies have been used to treat diseases caused by MDR without causing drug resistance, such as sonodynamic therapy (SDT), chemotherapy (CDT), photodynamic therapy (PDT), and photothermal therapy (PTT) [12,13,14,15]. Among them, PTT has been widely used in studies of combating resistant microbial infections due to its advantages of non-invasiveness, low toxicity, simple operation, broad-spectrum antimicrobial properties, and non-proneness to developing resistance [16,17]. PTT refers to the method by which a photothermic agent (PTA) converts light into heat under the irradiation of a light source and effectively kills pathogens through thermal effects, such as destroying the cell structure, and denaturing macromolecules, such as proteins and DNA [18,19,20]. The light source of PTT is usually near infrared light (NIR), because most of the biological chromophobe groups have strong absorption in the NIR region and can generate heat through non-radiative transition [21]. In addition, compared with ultraviolet-visible light (UV-Vis), NIR light can achieve deeper tissue penetration without causing damage to tissues [22,23]. The most commonly used wavelength of NIR light is 808 nm, because it can penetrate to a depth of 10 mm [24,25]. NIR can be further divided into NIR-I (700–900 nm) and NIR-II (900–1700 nm) [26]. In recent years, research on NIR-II agents has been increasing gradually, because NIR-II has a strong ability to penetrate into deep tissues; furthermore, the nanomaterials with absorption in the NIR-II region have low spontaneous fluorescence and a high signal-to-noise ratio in comparison with those in visible light and the NIR-I range [26,27,28]. Jiang et al. [29] prepared a PTA with bimodal absorption at both the NIR-I and NIR-II windows and found that the maximum permissible skin exposure limit was 0.3 W·cm^−2^ at NIR-I (808 nm) and 1 W·cm^−2^ at NIR-II (1064 nm). Therefore, PTAs in the NIR-II window have a broader prospect in the biomedical field.

At present, many materials have been found to have excellent photothermal conversion capabilities, such as metal materials [13,30], carbon-based materials [31,32,33], metal sulfides [34,35], and organic materials [36,37], which have been widely used in the fields of solar cells, bioimaging, and antibacterial and anti-tumor applications [38,39,40,41]. In clinical applications, the ideal PTAs should meet the following requirements [28,42,43]: (1) good biocompatibility and degradability without causing harm to the body; (2) high photothermal conversion efficiency (PCE), good light stability, and resistance to being photobleached; and (3) the temperature should be controlled below 55 °C, which can be highly antibacterial and will not cause tissue damage. PTAs can be divided into inorganic materials and organic materials. Common inorganic materials include Ag nanomaterials and carbon nanotubes. For example, Liu et al. [44] embedded gallic-acid-modified Ag nanoparticles into hydrogels, which, in synergy with the biological toxicity and photothermal effects of Ag itself, has a broad spectrum and efficient antibacterial performance, which can effectively combat chronic wounds caused by microbial infection. Although inorganic materials have high PCE and light stability, they are generally more biotoxic, and, in particular, they place a certain burden on the liver and spleen, which limit their application in biomedicine [45,46].

Organic conjugated materials are easy to synthesize and can be modified to have ideal properties and low biological toxicity; as such, they have a large development space in clinical applications [45,47,48]. Conjugated materials have a conjugated structure (an alternating composition of π and σ-bonds) and a delocalized electronic structure [49]. When conjugated materials are doped by oxidation, electrons in delocalized orbitals may migrate, resulting in a reduced band gap and a redshift of the absorption peak to obtain NIR response characteristics [50]. In addition, a great number of conjugated nanomaterials with excellent photothermal conversion efficiency (PCE), good photostability, and biodegradability are developed to be used in PTT [51,52,53,54]. According to the molecular weight, conjugated materials can be divided into conjugated small molecules and conjugated polymers. In 1974, Takeo Ito et al. [55] prepared polyacetylene films containing conjugated structures by using the Ziegler catalyst, thus opening the curtain on the study of conjugated polymers. In addition, pseudo-conjugated polymers have also been prepared to solve the problem that conjugated polymers are difficult to degrade.

Herein, PTT based on conjugated nanomaterials, including conjugated small molecules, conjugated oligomers, conjugated polymers, and pseudo-conjugated polymers, and its application in the fight against microbial infection in recent years are reviewed. The conjugated nanomaterials that can be used to prepare photothermal agents are summarized in Table 1. The synergistic antimicrobial therapy of PTT combined with PDT is also included. In addition, the research status, limitations, and prospects of photothermal therapy are discussed.

## 2. PTT Based on Conjugated Nanomaterials

### 2.1. Conjugated Small Molecules

Conjugated small molecules have a determined chemical structure with good reproducibility, easy metabolism, and high biocompatibility [56,57]. Commonly used commercial organic conjugated small molecule materials for PTT are indocyanine green (ICG) [58,59] and methylene blue (MB) [60,61]. However, their poor photothermal stability, ease of being photobleached, poor water solubility, and aggregation through hydrophobic interaction have greatly limited their application [45]. To encapsulate these molecules in amphiphilic substances is a useful solution. Ding et al. [62] wrapped ICG in water-soluble pillar 5 arene (WP5) through host–guest interaction to improve its stability. In addition to the dyes, conjugated small molecules with a donor (D)–acceptor (A) structure are also used in the preparation of PTAs. Lu et al. [63] constructed a ring conjugated by small molecule nanoparticles (Y6 NPs) based on an A-DA ′D-A structure, and under NIR irradiation of 808 nm at the influence of 1 W·cm^−2^, Y6 NPs exhibited excellent light stability and good PCE (57%), which can be used for efficient photothermal inactivation of pathogenic microbes.

**Figure 1:**
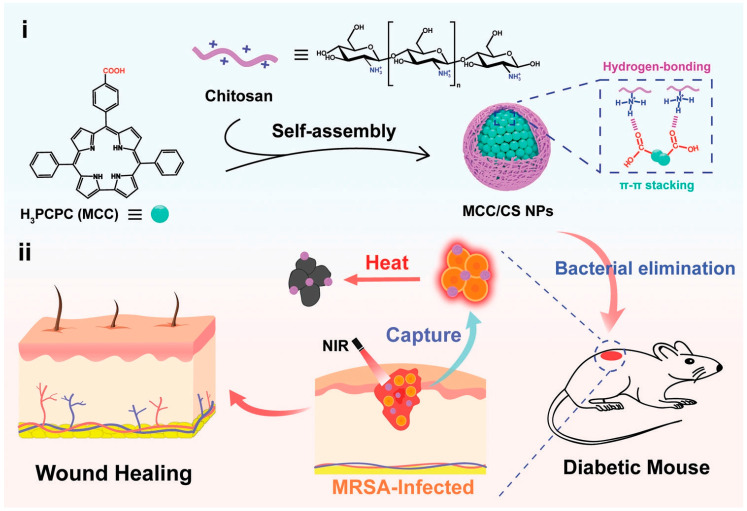
Schematic illustration of the preparation and antibacterial application of MCC/CS NPs. (**i**) Self-assembly of MCC/CS NPs from MCC and chitosan driven by hydrogen bonding and π–π stacking interactions and (**ii**) as a wound dressing to treat MRSA-infected diabetic mice wounds. Reprinted with permission from ref. [68]. Copyright 2022 Wiley-VCH GmbH.

Corrole is a tetrapyrrole macrocyclic compound of the porphyrin family containing an 18π-conjugated electronic structure, which has great potential in the preparation of PTAs due to its excellent photophysical properties and its in vivo metabolic rate [64,65,66,67]. However, its low water solubility and easy aggregation in water limit its application in clinical medicine. Yu et al. [68] synthesized 10-*p*-carboxyphenyl corrole (MCC) and self-assembled it with chitosan (CS) through hydrogen bonding and π–π accumulation to obtain an MCC/CS NPs core–shell structure, which was used to capture bacteria and to achieve efficient photothermal sterilization to promote wound healing (Figure 1). MCC is highly aggregated to form an intermediate nucleus through π–π accumulation and hydrophobic interaction. Frequent intermolecular collisions induce fluorescence quenching, thus enhancing the photothermal effect. The amino group in the hydrophilic CS forms hydrogen bonds with the carboxyl group on the CS, which wraps the MCC core as a hydrophilic shell to improve the solubility of MCC and thus improve the biocompatibility. Due to the close packing of MCC, the absorption peak of MCC/CS NPs has a bright redshift with a strong absorption in the range of 600–700 nm, which makes photothermal conversion more feasible. At room temperature, after 5 min of irradiation with a 500 mW·cm^−2^ laser at 660 nm, the NPs aqueous solution with a concentration of 50 μg/mL could be heated to 65.8 °C, and the PCE could reach 66.4%. After five ON/OFF cycles, the temperature of the NPs solution after irradiation was almost constant, indicating excellent photothermal stability. The photothermal effect of NPs is dependent on drug concentration and light power density, and therapeutic controllability can be achieved by changing these conditions. Under the same conditions, although the MCC alone can also warm up to more than 60 °C, the temperature will drop by nearly 20 °C after five ON/OFF cycles. The amino group on the CS shell gives MCC/CS NPs positive charges, which can trap negatively charged bacteria through electrostatic interaction. After further NIR laser irradiation, NPs generate heat and destroy the structure of bacteria, which causes the loss of cell contents through thermal effects, and the in vitro inhibition rate for *E. coli* and MRSA is nearly 100%. In a mouse wound model infected with MRSA, the wound temperature rose to 51.3 °C under NIR irradiation. On the third day of treatment, the bacteria at the wound were counted, and the survival rate of bacteria after NPs+L treatment was 10–20%, which was significantly lower than that of other controls. On day 13, the wound area of the mice was only 1.7%. Histological analysis showed that the inflammation in the NPs+L group was significantly inhibited on day 4. On day 14, hair follicles, blood vessels, and connective tissue structures were well regenerated with significant collagen deposition. In addition, the weight of the mice did not change significantly throughout the treatment. In conclusion, under NIR light, MCC/CS NPs can effectively inactivate bacteria, promote tissue regeneration, and have good biocompatibility, providing a possible strategy for the development of supramolecular photothermal antimicrobials and design inspiration for nanomaterials for the rapid and effective healing of diabetic wounds.

**Figure 2:**
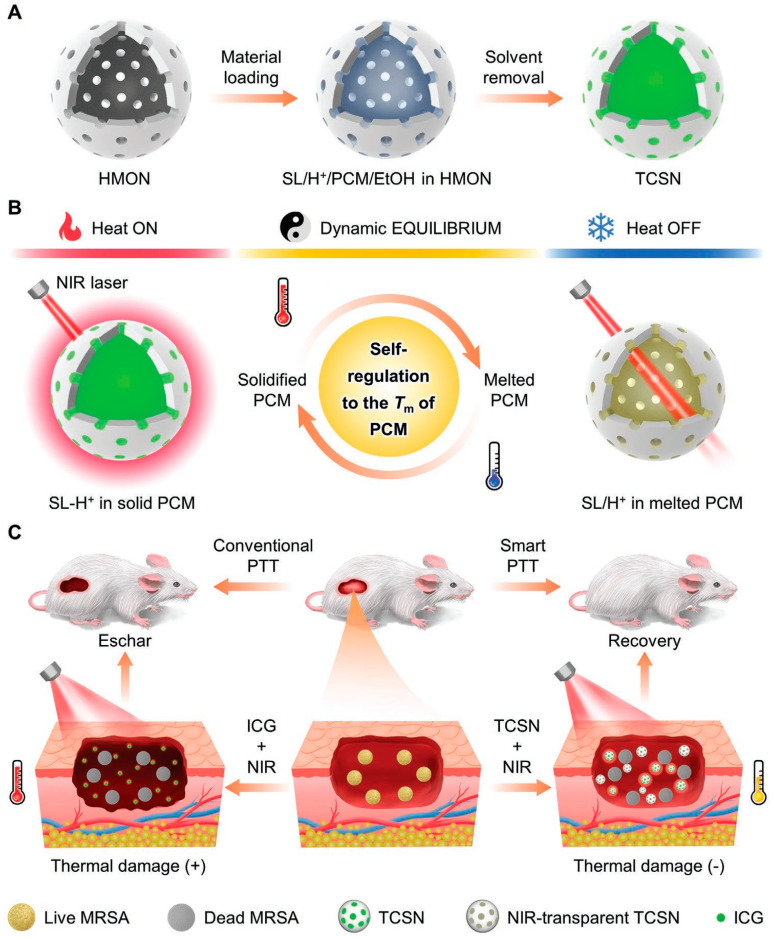
(**A**) Fabrication and (**B**) smart temperature control mechanism of the smart photothermal nanoplatform. (**C**) Comparison of the therapeutic effects between the smart PTT and conventional PTT. Reprinted with permission from ref. [74]. Copyright 2022 Wiley-VCH GmbH.

The temperature of most PTAs prepared using traditional methods is uncontrollable, and the temperature may be too high during treatment, resulting in irreversible thermal damage to healthy tissues [69,70,71,72]. At present, the commonly used solution is to change the experimental conditions, such as the light power density and the concentration of PTAs [73], but the immediacy of this method is relatively poor, and heat accumulation may occur and even cause serious burns and secondary infections. Therefore, it is necessary to develop a PTT platform that can accurately and quickly adjust the temperature. Wang et al. [74] designed a thermostatic intelligent photothermal platform using phase-change materials (PCM) for safely and efficiently combating microbial infections (Figure 2). Firstly, spironolactone (SL) with photothermal properties, saturated fatty acid myristate (MA), and proton donor bisphenol A (BPA) were blended to obtain ternary thermochromic materials. In the solid MA at low temperature, BPA provides protons to the transparent SL, causing the reversible conversion of SL to SL-H^+^ with NIR absorption in the range of 650–1000 nm, while the system changes to a dark green color. Under the irradiation of NIR, the photothermal effect of SL-H^+^ makes the temperature of the system rise, and the solid MA melts into a liquid. In liquid MA, SL-H^+^ loses a proton to become SL without a photothermal effect, and heat production stops. This process moves in cycles so that the temperature of the system is in a dynamic equilibrium near the melting point of the MA. The temperature-controlled intelligent nanomaterial (TCSN) was obtained by loading the PCM into a hollow silica nanomaterial (HMONs). The silanol group in HMONs can form hydrogen bonds with water, improving the solubility and dispersibility of the PCM in water. At the same time, the addition of organic groups reduced the silanol group density on the surface of the HMONs, thus decreasing the hemolysis rate and improving the biocompatibility. Triggering of the self-regulation mechanism requires reaching the MA melting point of 49 °C, which is affected by the concentration of SL and the laser power density. When the concentration of SL is 40 μg/mL, the maximum equilibrium temperature is not affected by the laser power density, and it is maintained at about 49 °C. When the laser power density is maintained at 1.5 W·cm^−2^, the maximum equilibrium temperature does not change with the SL concentration. When TCSN is placed in a water bath at different temperatures (27–37 °C), it will eventually reach 49 °C without further change, indicating that the inherent temperature control mechanism of PCM will not be affected by external factors. After five ON/OFF cycles, the equilibrium temperature of TCSN is almost unchanged, showing good photothermal stability. According to in vitro antibacterial experiments, the photothermal inhibitory rate of TCSN on MRSA reached 99.99% after 5 min of irradiation with the NIR laser, suggesting that the inhibitory mechanism was mainly high-temperature-mediated protein denaturation. In the MRSA mouse infection model, under the same light and dose conditions, ICG rapidly heated up to 63 °C within 2 min and then was gradually photobleached and decreased to 50 °C within 5 min. TCSNs rose steadily within 5 min and remained at about 50 °C. On day 10 of the treatment, the wounds in the TCSN+NIR group were almost completely healed, while in the ICG+NIR group, the wound was further enlarged due to the formation of a heat-induced scab on the 4th to 6th day. Under the conditions of laser irradiation, although the bacteriostasis rate in the ICG group (99.99%) was higher than that in the TCSN group (99.9%), the sudden heat burst also brought serious side effects and hindered wound repair. According to H&E staining, ICG without temperature control can produce local heat damage, causing tissue necrosis and increased inflammation. TCSN can not only effectively fight bacteria but also reduce inflammation and promote tissue regeneration and wound repair. In this study, a constant temperature photothermal platform with NIR response was established, which has great application prospects in the safe and effective treatment of bacterial infections.

**Figure 3:**
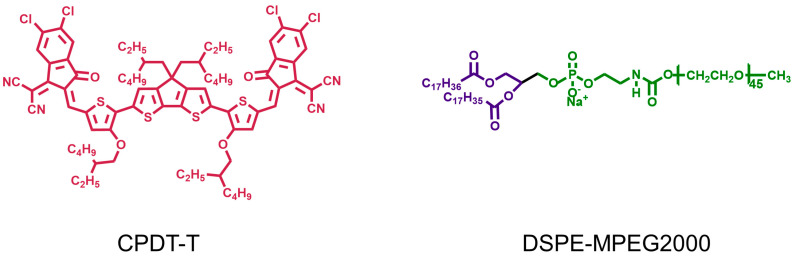
The structure of CPDT-T and DSPE-MPEG2000.

Most of the excitation light of conjugated small molecule PTAs is located in the NIR-I region, which greatly limits its application. Jia et al. [56] developed an NIR-II-triggered photothermal platform based on the acceptor–donor–acceptor (A-D-A) structure for combating microbial infection (Figure 3). Firstly, conjugated small molecule CPDT-T was constructed to reduce fluorescence emission efficiency by enhancing intramolecular charge transfer (ICT) in the A-D-A structure, thus improving the photothermal conversion ability. Then, nanoreagents (CNPs) were obtained by assembling the amphiphilic polymers DSPE-MPEG2000 and CPDT-T through hydrophobic interaction. CNPs have strong wide absorption in the region above 1000 nm with NIR-II luminescence. This is due to the fact that CPDT-T has a narrow band gap of only 1.36 eV, which is conducive to the redshift of the absorption peak. When irradiated with a laser at 1064 nm, the PCE of CNPs was determined to be 49%. The photothermal effect of CNPs is dependent on laser power density and concentration, which can regulate the wound temperature to avoid unnecessary thermal damage. When the concentration of CPDT-T was 40 mg/mL, the inhibition rate of CNPs on *S. aureus* reached almost 100% under the irradiation of the NIR laser at 1064 nm with an intensity of 1.2 W·cm^−2^ for 8 min. After five ON/OFF cycles, it can be seen that CNPs have excellent photobleaching resistance. This method solves the problem that conjugated small molecules are rarely used in the NIR-II region and broadens the application prospects. Furthermore, the absorption of PTAs could be regulated by varying the acceptor structure in conjugated molecules to be broadened in the NIR-II region. Shao et al. [75] used trianiline (TPA) as an electron donor and benzo [1,2-c:4,5-c0] bis ([1,2,5] thiadiazole) (BBT) as an acceptor to synthesize the D-A-D typeconjugated small molecule (CSM2) in response to NIR-II. With the increase in the number of thiophene bridges from 0 to 2, ΔEst effectively decreased to 1.06 eV, and the redshift of the absorption curve gradually redshifted to the NIR-II region with a PCE of 31.6%.

### 2.2. Conjugated Oligomers

Conjugated oligomers with photothermal conversion capacity are generally obtained by conjugation of a limited number of aromatic rings. The common strategies to improve the photothermal properties of conjugated oligomers are to introduce the donor–acceptor (D-A) structure, improve the intramolecular charge transfer (ICT), and reduce the band gap [75]. Tang et al. [76] formed a dark twisted intramolecular charge transfer state by linking the alkyl group to the D-A center, which restricted the intermolecular interaction and maintained the intramolecular motion in the aggregation state, thereby increasing the PCE from 20.7% to 31.2%. Wu et al. [77] designed a PTA-containing D-A structure based on conjugated borodipyrrole methylene (BODIPY) oligomers. The interaction between molecules was inhibited by large trimethyl groups, and the excited BODIPY motif moved around the vinyl group in the aggregate state, achieving a high PCE of 72.4%. Jia et al. [78] designed three conjugated oligomers with excellent photothermal properties by introducing an acceptor with a strong electron-withdrawing ability into the A-D-A structure. Among them, BTP-4F with two phenyl-fluorinated cohesive groups had a PCE of 83%, which was higher than most of the conjugate oligomers the PTA reported, and the inhibition rate against Amp^r^ *E. coli* was 99.9% at a low concentration (10 μg/mL) and light dose (808 nm, 550 mW·cm^−2^).

Water solubility often determines the biocompatibility and feasibility of the biological application of conjugated molecules [79]. Except for the photothermal nanoparticles that could be dispersed in water, conjugated PTAs could be modified with a hydrophilic component to improve water solubility. Water-soluble conjugated oligomers are usually composed of two parts, a hydrophobic conjugated main chain and a hydrophilic branch chain, which could be a neutral polyethylene glycol (PEG) chain or an alkyl with a positively charged quaternary ammonium/quaternary phosphorus salt end [80]. He et al. [81] synthesized a water-soluble PTA (PCE = 51%) modified with an ammonium group, which could trap negatively charged bacteria through electrostatic interaction and destroy the stability of the membrane. Under NIR light irradiation (808 nm, 1 W·cm^−2^, 10 min), the survival rate of both MRSA and *E. coli* treated with PTA was only 1%. Li et al. [82] modified a nano-photothermal agent containing an A-D-A structure with PEG to obtain a highly water-soluble PTA with a PCE of 82%, which is not easy to aggregate and can be degraded by endogenous enzymes.

**Figure 4:**
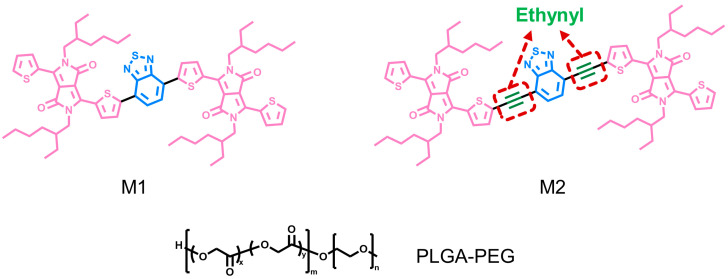
The structure of M1, M2, and PLGA-PEG for the preparation of conjugated nanomaterials.

Zhang et al. [83] promoted PCE by introducing acetylene groups into a conjugated framework for wound treatment of bacterial infections (Figure 4). First, the conjugated oligomer M1 was prepared using divinyldiketopyrrole and benzo [c][1,2,5] thiadiazole (BTD), and then the acetylene group between benzothiazole and thiophene was introduced into M1 to obtain M2. M1 and M2 do not emit fluorescence, indicating that the energy is mainly dissipated in a non-radiative way and that it has a great photothermal conversion ability. At 642 nm, the mass extinction coefficients of M1 and M2 are 16.19 L·g^−1^·cm^−1^ and 36.32 L·g^−1^·cm^−1^, respectively, implying that the introduction of the acetylene group improves the extinction coefficient and thus the light absorption capacity of M2. In order to further use M1 and M2 in biological systems, hydrophilic nanoparticles M1-NPs and M2-NPs were prepared by nanoprecipitation with PLGA-PEG. The absorbance of M2-NPs is significantly higher than that of M1-NPs when the concentration is 10 μg/mL in 600–700 nm. The calculation shows that the extinction coefficient of M2-NPs (30.26 L·g^−1^·cm^−1^) is twice that of M1-NPs (15.34 L·g^−1^·cm^−1^), and the light absorption capacity is still much greater than that of M1-NPs. When the laser at 660 nm with an intensity of 750 mW·cm^−2^ is irradiated for 8 min, the M2-NPs solution can be heated to about 60 °C with a temperature change of 30 °C, while the temperature change of M1-NPs is only 12 °C. If M1-NPs want to reach 60 °C at the same condition, the required concentration is 20 μg/mL, which is twice that of M2-NPs, indicating that the method of introducing acetylene groups into the conjugated framework can enhance the photothermal performance by improving the light absorption capacity. In addition, after five ON/OFF cycles, M1-NPs and M2-NPs have strong light stability and excellent PTT potential. After 8 min of laser irradiation at 660 nm with an intensity of 750 mW·cm^−2^, M2-NPs killed 97% of *E. coli* and *S. aureus* at a concentration of only 7 μg/mL, while M1-NPs required 13 μg/mL to achieve the same results. To kill 99% of *C. albicans*, the concentrations required for M1-NPs and M2-NPs are 18 μg/mL and 10 μg/mL, respectively. Their antimicrobial mechanism is to denature proteins and DNA through a high temperature and to increase membrane permeability, thereby causing cell contents to flow out. These two NPs had broad-spectrum antimicrobial activity, among which M2-NPs had significantly stronger antimicrobial activity than M1-NPs, so M2-NPs were selected for the characterization of their clinical application potential. After laser irradiation, the temperature of a wound with M2-NPs injection increased to 52 °C, and the infected wound was completely healed on the 10th day, with low cytotoxicity and organ toxicity. This study proposed a method to improve the light absorption capacity by introducing an ethynyl group into conjugated molecules, which provided a new idea for enhancing the photothermal properties of conjugated nanomaterials.

**Figure 5:**
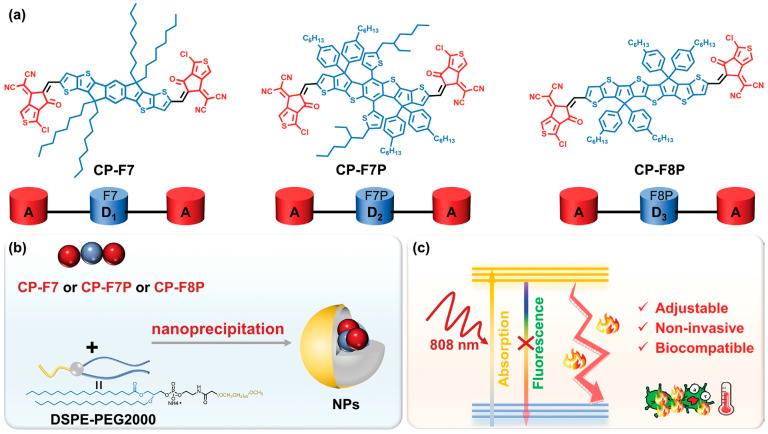
Illustration of (**a**) the chemical structures of conjugated oligomers, (**b**) the preparation of NPs, and (**c**) photothermal antimicrobial therapy based on NPs. Reprinted with permission from ref. [84]. Copyright 2023 Wiley-VCH GmbH.

Yuan et al. [84] designed three tunable conjugated oligomers with A-D-A structures for the efficient treatment of microbially infected wounds (Figure 5). CP-F7 and CP-F7P containing seven conjugated rings and CP-F8P containing eight conjugated rings in the donor structure were prepared, and then the corresponding spherical nanoparticles were prepared by coprecipitation with amphiphilic polymer DSPE-PEG2000. All three nanomaterials have strong wide absorption in the NIR range of 500–900 nm, among which CP-F8P NPs also show obvious redshift in the NIR range of 800–900 nm. Under the irradiation of an 808 nm laser with a fluence of 1 W·cm^−2^, the temperatures of CP-F7 NPs, CP-F7P NPs, and CP-F8P NP aqueous dispersions elevated rapidly and displayed variation values of 32.6, 38.4, and 53.6 °C after 4 min, respectively. The strong electron donating ability of the donor in CP-F8P, which enhances the intramolecular charge transfer (ICT), improves the photothermal conversion capacity dramatically, with a high PCE of 81.6%. After five ON/OFF cycles, the temperature distribution is almost unchanged, indicating that CP-F8P NPs have good thermal stability. In vitro antimicrobial experiments showed that CP-F8P NPs had almost no dark toxicity to microorganisms. However, after 5 min of irradiation with an 808 nm laser (550 mW·cm^−2^), the bacteriostatic rate of 14 μg/mL CP-F8P NPs on Amp^r^ *E. coli* was 99.3%, and the bacterial suspension was heated to 47.8 °C. When the concentrations were 10 μg/mL and 12 μg/mL, the killing efficiencies toward *S. aureus* and *C. albicans* reached more than 99%. SEM observation showed that the structure of the bacteria was damaged and that the nucleic acid contents were leaked after photothermal treatment. These results indicate that CP-F8P NPs have high and broad-spectrum photothermal antimicrobial activity. When 20 μg/mL of CP-F8P NPs was added to the wounds of MRSA-infected mice, the wound tissue temperature rose to 49.5 °C after irradiation for 5 min, and this temperature is enough to treat the infection without causing damage to healthy tissue. On the 9th day of treatment, the wound healing rate was more than 70%, which is much higher than that of other controls (<30%). In addition, CP-F8P NPs could not lead to inflammation in mouse tissues and toxicity toward organs and blood, indicating that CP-F8P NPs can effectively accelerate the healing of infectious wounds. This study improves photothermal performance by adjusting the donor structure in A-D-A-type conjugate oligomers, which provides an alternative strategy for designing PTAs with superior performance.

### 2.3. Conjugated Polymers

#### 2.3.1. Polyaniline (PANI)

Polyaniline (PANI) was first reported as a conjugated polymer for PTT [22]. In 1826, aniline monomer was isolated by pyrolysis of indigo, and later studies obtained PANI through polymerization reaction [85]. Polyaniline has good photothermal properties, biocompatibility, and electrical conductivity, but low solubility, which greatly limits its application [85,86,87]. To improve the water solubility and photothermal performance of PANI, doping and composite material preparation are generally used methods. For example, polymer-F127-containing polyoxyethylene chains could be used to wrap PANI to form water-dispersed PANI NPs for PTT [88]. After doping PANI, the absorption peak appears redshift, which is due to the fact that protonated dopants (such as strong acids and transition metals) create a band gap between the valence and conduction bands, inducing electron motion and reducing the excited energy level [89]. Ghahremanloo et al. [90] prepared doped polybenzenediamine by oxidative polymerization, which significantly improved its antioxidant and photothermal properties. Yang et al. [91] transformed PANI from a purple emeralidine base (EB) to a dark green emeralidine salt (ES) state by doping, thereby preparing PTA with excellent photothermal properties.

**Figure 6:**
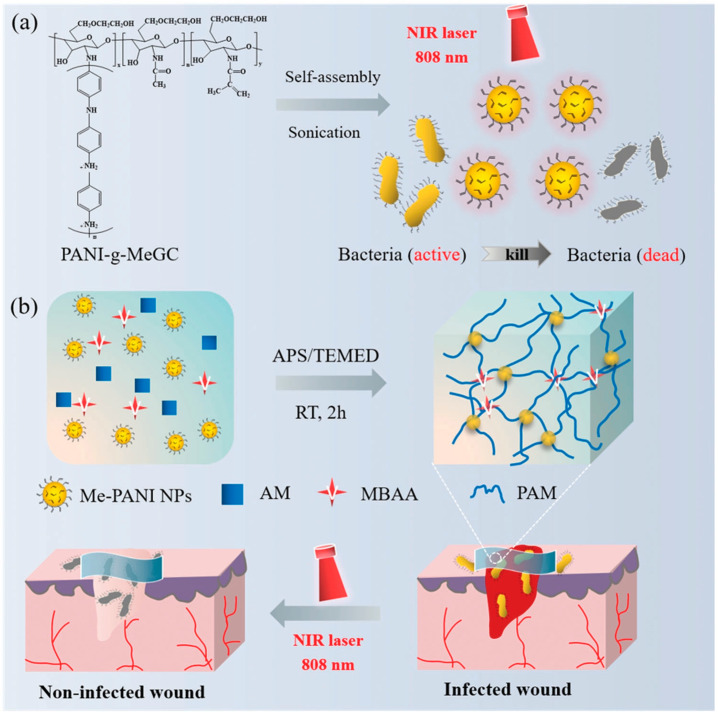
(**a**) Self-assembly of PANI nanoparticles (Me-PANI NPs) and their photothermal antibacterial illustration upon NIR laser irradiation. (**b**) Schematic diagram of the preparation of the NPs@PAM hydrogel and its potential application as a wound dressing. Reprinted with permission from ref. [92]. Copyright 2021 Wiley-VCH GmbH.

Pang et al. [92] innovatively designed a photothermal hydrogel using PANI NPs as a crosslinking agent for effective antibacterial action and to prevent the diffusion of PTA to surrounding healthy tissues (Figure 6). The solubility of PANI was improved by grafting hydrophilic MeGC, and then Me-PANI NPs were obtained by self-assembly in an aqueous solution. Me-PANI NPs have a maximum absorption peak of about 600 nm and a wide absorption in the NIR region. After irradiation with an 808 nm laser, the temperature rose sharply within the first 5 min and reached a stable state after 10 min. The heating capacity of Me-PANI NPs is concentration-dependent, and when the concentration is greater than 1 mg/mL, the temperature can reach more than 50 °C. Me-PANI NPs have no dark toxicity, and NPs at concentrations of 1 mg/mL and 2.5 mg/mL can reduce *S. aureus* by two and seven orders of magnitude after laser irradiation, respectively. At the same time, Me-PANI NPs+NIR was able to clear most MRSA biofilms. Me-PANI NPs were crosslinked with polyacrylamide (PAM) to obtain NPs@PAM hydrogel. The addition of NPs can effectively dissipate the external energy and greatly improve the mechanical properties of PAM hydrogel, with a high swelling rate of 1300%, and the hydrogel can be stretched up to four times longer without damage. After the hydrogel was placed at 37 °C for 7 days, the weight retention rate was 69.6 ± 2.86 wt %. After laser irradiation for 10 min, the weight was 73.1 ± 1.18 wt % of the original weight, indicating that the hydrogel could maintain the humidity of the wound environment. When the concentration of NPs increased to 10 mg·mL^−1^, the surface temperature of the hydrogel reached 50.6 °C after irradiation with an NIR laser for 3 min. And, it rose to 58 °C at 10 min, at which point virtually all *S. aureus* and *E. coli* could be killed. The NPs@PAM hydrogel was cultured with NIH 3T3 cells for 24 h, and the cell survival rate was over 80%, showing good biocompatibility. The obtained photothermal hydrogel can realize the controllable treatment of bacteria-infected wounds.

**Figure 7:**
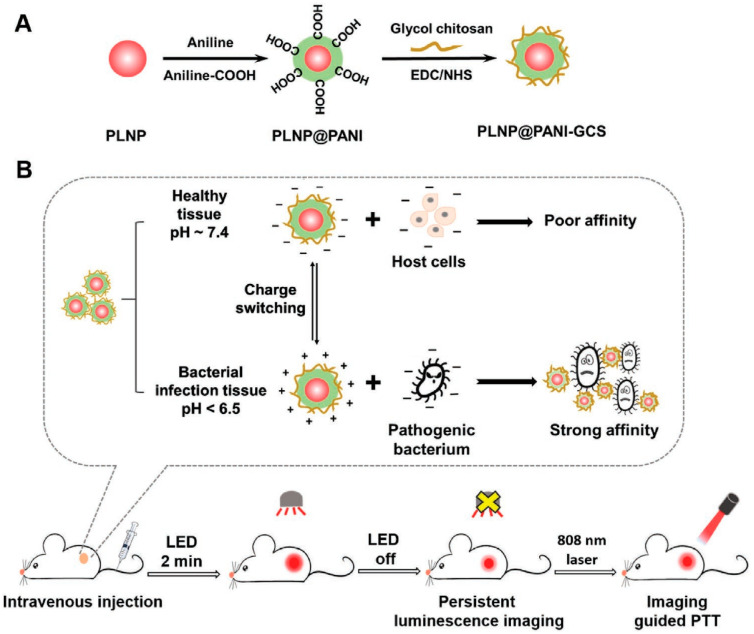
Schematic illustration of (**A**) the preparation of PLNP@PANI-GCS and (**B**) its persistent imaging-guided photothermal therapy for bacterial infection. Reprinted with permission from ref. [24]. Copyright 2023 Wu, Huang, Huang, Wang, and Wei.

Yan et al. [24] designed a visualized pH-responsive photothermal nanoplatform for precise and efficient antibacterial treatment (Figure 7). Core-shell PLNP@PANI nanoparticles were prepared by oxidation polymerization of aniline and aniline-COOH using PLNPs as the core and PANI as the shell. Then, glycol chitosan (GCS) was modified by amidation reaction to obtain PLNP@PANI-GCS with targeting ability. PLNPs can be activated to luminescence under red LED light to achieve long-term sustainable fluorescence imaging for 48.5 h for visualizing and guiding treatment. Under physiological conditions of pH 7.4, PLNP@PANI-GCS exhibit a zeta potential of −4.7 mV with a small amount of negative charge, which will not cause damage to healthy cells. However, it contains positive charge in the acidic microbial infection environment (pH 6.5) with a zeta potential of 19 mV, so it can target and be concentrated at the infection site through electrostatic interaction. After irradiation with an 808 nm NIR laser with an intensity of 1.5 W·cm^−2^ for 8 min, the temperature of PLNP@PANI-GCS with a concentration of 1 mg/mL can rise to 48.4 °C at pH 7.4. Furthermore, when the pH was 6.5, the maximum temperature increased by 12.6 °C. This is due to the charge transfer between the quinone-type ring and the benzene-type ring of PANI and the absorption peak redshift when pH decreases. Therefore, in an acidic environment, the PCE of PANI is enhanced, which can produce a stronger photothermal effect, but it will not cause photothermal damage to normal tissues in a physiological environment. In vitro antibacterial experiments were carried out in PBS solution at pH 6.5, and 1 mg/mL of PLNP@PANI-GCS showed over 99% antibacterial efficiency for *E. coli*, *S. aureus*, and MRSA under NIR irradiation. PLNP@PANI-GCS was injected intravenously into MRSA-infected mice, and fluorescence appeared in the abscess area 24 h later, with the strongest intensity on days 2–3. After 5 min of laser irradiation, the temperature of the abscess reached 52.2 °C, and no colony was found by the plate counting method. As the infection and inflammation improved, the fluorescence gradually disappeared on day 6. On the 6th day of treatment, there was almost no inflammatory infiltration at the wound site, and the tissue regeneration was good. Within 10 days of injection, 71.5% of NPs was excreted in the stool and not absorbed by the body. This method provides a good reference for the design of an intelligent targeted antibacterial platform.

#### 2.3.2. Polydopamine

Polydopamine (PDA) is formed by the spontaneous oxidation and polymerization of dopamine (DA) under alkaline conditions [93,94]. The indole-5,6-quinone (IQ) in PDA is a conjugated structure, and the D-A structure is formed between the 5,6-dihydroxyindole (DHI) and IQ units, so it has photothermal conversion performance in the NIR range [95,96]. There are many active functional groups on PDA with good photothermal properties and biocompatibility, so it is easily modified to be used in PTT [96,97,98,99,100]. When using simple PDA as the PTA, there are some problems, including easy aggregation, which results from a large number of active groups, thereby reducing light absorption efficiency, and uneven heat distribution to bacteria [101]. Moreover, single PDA has a relatively low PCE in the NIR region [102]. Qi et al. [103] grew Ag in situ on the surface of PDA NPs to obtain PDA@Ag NPs, and then combined it with a cationic guar gum (CG) hydrogel network through physical interactions, such as hydrogen bonding and electrostatic force, to obtain CG/PDA@Ag hydrogel, leading to an increase in PCE from 16.6% to 38.2%. And, the inhibition rates of CG/PDA@Ag hydrogel toward *S. aureus* and *E. coli* were 99.8% and 99.9%, respectively, under NIR irradiation (808 nm, 1 W·cm^−2^, 3 min). Similarly, Li et al. [104] prepared PDA@Cu NPs by growing Cu in situ on the surface of PDA, and then dispersed it evenly in hydrogel, resulting in PCE increasing from 30.8% to 54.2% and showing broad-spectrum antibacterial activity under NIR irradiation (808 nm, 1 W·cm^−2^, 5 min) with inhibition rates for *S. aureus* and *E. coli* of 98.2% and 100%, respectively.

**Figure 8:**
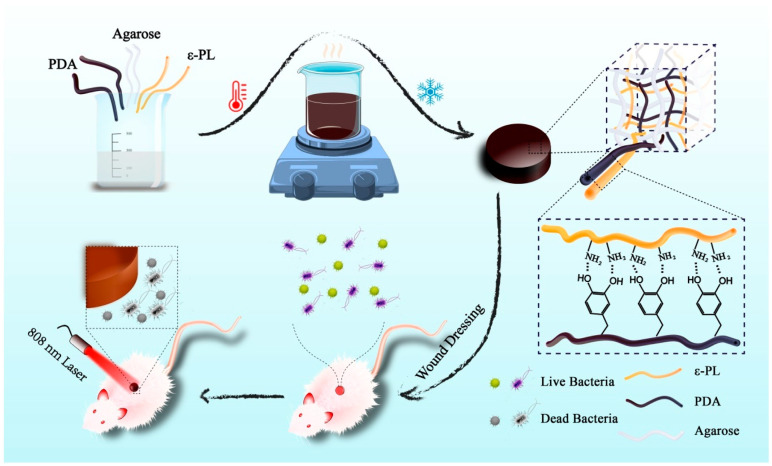
Illustrated preparation of ADPH for bacteria-infected wound healing. Reprinted with permission from ref. [105]. Copyright 2021 Elsevier Ltd.

However, when the PDA is loaded on the hydrogel, the stability is usually poor, and it is easy to escape from the hydrogel. Qi et al. [105] designed a functional stable photothermal hydrogel dressing for combating wound infection (Figure 8). Using ε-polylysine (ε-PL) as the stabilizer, the PDA was stably embedded in an agar–hydrogel matrix to obtain agarose/PDA/ε-PL hydrogel (ADPH). ADPH has good elasticity and pressure resistance and a good swelling rate, which can provide an ideal healing environment for wounds and effectively promote wound repair under the synergistic action of photothermal antibacterial treatment. When the PDA concentration was 2 mg/mL, the PCE of ADPH was 63.9%. When irradiated with a laser at 808 nm for 5 min, the temperature of the ADPH was dependent on the laser power density, so the temperature could be controlled by adjusting the light intensity. After four cycles of irradiation with 1 W·cm^−2^ of NIR, the temperature of the ADPH was maintained near 45 °C, while the temperature generated by the ADH in the absence of ε-PL was reduced from 44.6 °C to 28.9 °C, indicating that ε-PL can make PDA stable in hydrogel through electrostatic interaction. ADPH shows good cytocompatibility and blood compatibility, suggesting great potential for clinical application. When irradiated with an 808 nm NIR laser for 10 min, the antibacterial rates of *S. aureus* and *E. coli* reached nearly 100% regardless of whether the ADPH was soaked in PBS for 24 h. The ADH did not show obvious antibacterial activity after soaking, and the PDA was gradually lost without ε-PL binding. The inhibitory mechanism of ADPH is to destroy the structural integrity of the bacterial cell membrane and cell wall. In the full-layer infection wound model of the dorsal skin in mice, after 10 min of NIR irradiation, the wound temperature of the ADPHi (immersed in PBS for 24 h) treatment rose to 59.4 °C, and almost no bacteria survived in the wound. During 11 days of treatment, the wound healing rate of ADPHi treatment in the presence of the NIR laser was much higher than that of the control group. On day 11, this group had the smallest scar width (1.49 mm), less inflammation, and large collagen deposition. In this study, the PDA was firmly bound in agar–hydrogel with ε-PL, and stable photothermal properties and good physicochemical characteristics were obtained, which provided an important reference for the management of infectious wounds.

**Figure 9:**
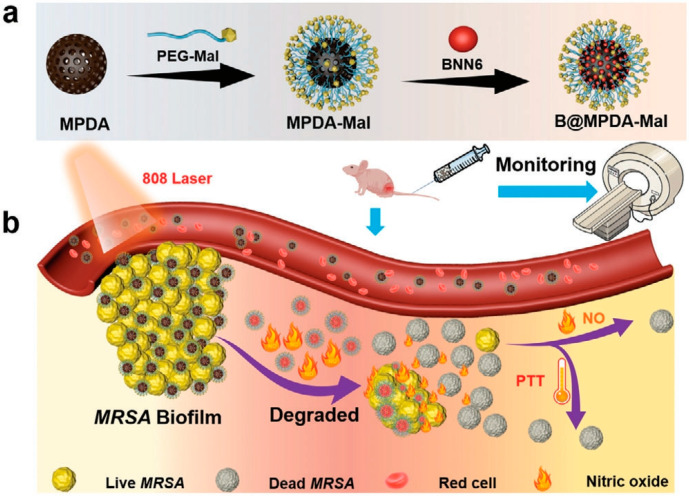
(**a**) Illustrated preparation of B@MPDA-Mal. (**b**) Schematic illustration of the B@MPDA-Mal nanoplatform, which precisely targets antibacterial infection. Reprinted with permission from ref. [107]. Copyright 2023 Wiley-VCH GmbH.

Wounds are susceptible to microbial infection and become difficult to heal, so early detection and removal of pathogens is particularly important [106]. Lv et al. [107] designed a platform capable of detecting microbial infection, accurately targeting the site of bacterial infection, and effectively killing bacteria under the synergistic action of PTT and NO (Figure 9). Dopamine was self-polymerized in an ammonia solution to obtain spherical mesoporous polydopamine (MPDA) with dense surface pores and then coupled with polyethylene glycol modified with maltotriose (PEG-Mal) by Schiff base and Michael addition reaction to obtain MPDA-MAL. Finally, NO donor BNN6 was loaded to obtain the intelligent antibacterial platform B@MPDA-Mal. After 5 min of irradiation with an 808 nm laser, the temperature of the MPDA solution increased from 26.2 °C to 67 °C. Under the same conditions, B@MPDA-Mal can be increased by 36.8 °C, and the warming trend is the same, indicating that PEG and BNNA have no obvious influence on the photothermal performance of MPDA. The photothermal properties of B@MPDA-Mal improve with the increase in drug concentration and laser power density. After five ON/OFF cycles, B@MPDA-Mal still showed good photostability, indicating that this nanoplatform has good potential for PTT application. In a double-infected mouse model simulating aseptic and bacterial inflammatory infection, fluorescence began to appear at the site of bacterial infection after 3 h of intravenous administration of B@MPDA-Mal, and fluorescence remained at the 24th hour. The maximum accumulation time of fluorescence at the infection site was 6 h for *E. coli* and *S. aureus* and 12 h for MRSA. There was no fluorescence in the wound caused by lipopolysaccharide-induced aseptic inflammation, indicating that B@MPDA-Mal has bacteria-targeting capabilities. This is because maltotriose can selectively enter the bacterial interior under the action of the bacterial maltodextrin transporter and will not be absorbed by the human body. After 10 min of 808 nm laser irradiation, the number of bacteria treated with B@MPDA-Mal decreased by 92.3%, and the biofilm thickness decreased by 9.85 μm compared with the non-irradiation group, indicating that the drug has strong antibacterial and anti-biofilm activities under the synergistic effect of PTT and NO. Under laser irradiation, the photothermal effect produced by MPDA-Mal will cause the bacterial cell membrane to be damaged, while the NO released by BNN6 will enter the cell and undergo a nitrosation reaction with the DNA, thus promoting bacterial death. In a mouse model of MRSA-infected leg myositis, NIR+B@MPDA-Mal treatment reduced bacteria by 95% and effectively eliminated muscle abscesses by day 12 without biotoxicity. This nanoplatform achieves precise targeting of bacteria through maltodextrin, which solves the problem that traditional NO cannot be enriched at the infection site; at the same time, it achieves high antibacterial efficiency in combination with PTT, providing new insight for the treatment of deep drug-resistant bacterial infections.

#### 2.3.3. Polypyrrole

Polypyrrole (PPy) is formed by the oxidation polymerization of pyrrole in water with the oxidizing agent FeCl_3_ [108]. The transition between π and π* bonds in PPy gives it a strong and wide absorption in the NIR region and good photothermal properties [109,110]. PPy is also biocompatible and can be used for PTT [111]. However, the poor water solubility of PPy greatly limits its application, and its dissolution can be improved by modifying polar groups or by adding surfactants [112,113]. Alizadeh et al. [112] modified PPy with a dibasic acid as the dopant and stabilizer to improve its water solubility. Antony et al. [113] prepared water-soluble PPy NPs with various sizes (100–800 nm) using self-organized micelles of amphiphilic azobenzenesulfonic anionic surfactants.

**Figure 10:**
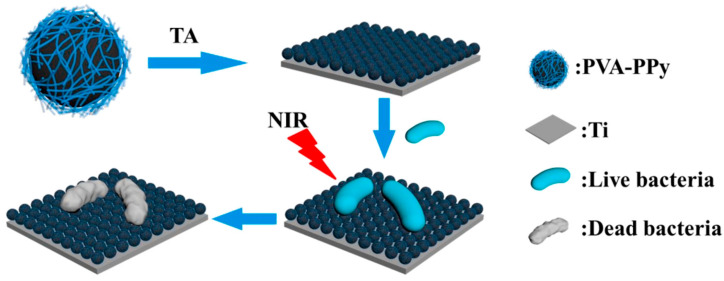
Preparation and photothermal antibacterial activity of the TPP coating. Reprinted with permission from ref. [114]. Copyright 2022 Elsevier B.V.

Wang et al. [114] designed a photothermal antibacterial coating for the preparation of medical implant materials (Figure 10). In the presence of FeCl_3_, a pyrrole monomer was polymerized in polyvinyl alcohol (PVA) micelles to obtain PVA-PPy NPs with strong and wide absorption in the NIR region. Then, PVA-PPy NPs were bonded with tannic acid (TA) through hydrogen bonding to obtain a TA/PVA-PPy coating, and Ti-TPP was obtained by co-deposition on the Ti surface through the inherent viscosity of TA. After irradiation with an 808 nm NIR laser, the temperature elevated rapidly in the first 4 min and remained stable with an increase of 27.6 °C. The photothermal inhibition rates of Ti-TPP for *S. aureus* and *E. coli* in vitro were 98.3% and 97.2%, respectively, due to the thermal effect of the coating on damaging the cell structure of bacteria under light. The hydrophilic PVA in the coating can form a hydration layer to reduce bacterial adhesion and effectively prevent the formation of a biofilm under the synergistic effect of photothermal antibacterial effects. This antibacterial coating prevents bacteria from sticking and kills bacteria efficiently, making it a potential candidate for medical implants.

**Figure 11:**
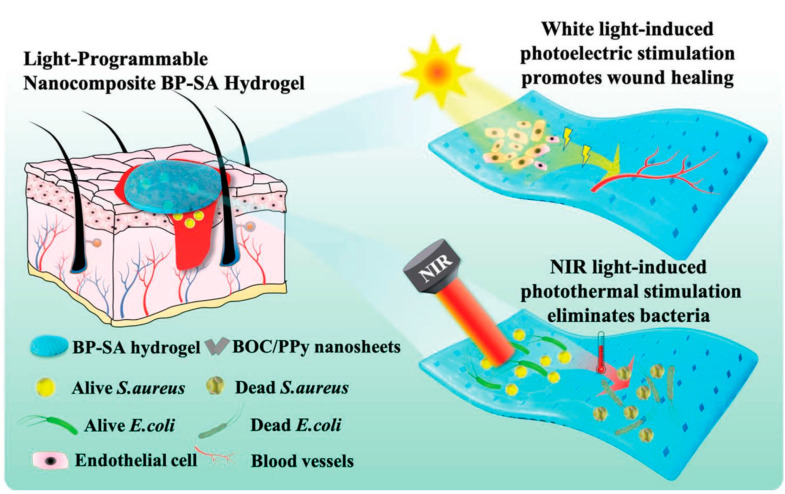
Illustration of the light-programmable BP-SA nanocomposite hydrogel for promoting wound healing and eliminating bacterial infection under white light and NIR light. Reprinted with permission from ref. [119]. Copyright 2022 Wiley-VCH GmbH.

Exogenous electrical stimulation at the wound site can promote wound tissue regeneration by mimicking or enhancing bioelectricity [115,116]. Under the synergistic effect of light and electricity, photoelectric response materials can not only effectively fight bacteria through phototherapy but also promote wound repair through electrical stimulation, further improving wound healing efficiency [117]. In addition, the three-dimensional network structure of hydrogels is similar to the extracellular matrix (ECM), which can provide a good healing environment for wounds [118]. Zhang et al. [119] designed a light-programmable composite hydrogel with good photoelectric and photothermal properties for eliminating microbial infection and promoting wound healing (Figure 11). BOC/PPy composite nanosheets with a thickness of 5 nm were constructed by in situ oxidative polymerization on the surface of BiOCl nanosheets. Due to the presence of conjugated structures in PPy, BOC/PPy has strong and wide absorption in the NIR region, a narrow band gap (2.80 eV), and a high photogenerated carrier separation ability. BOC/PPy nanosheets have high electron transport efficiency, which can promote the separation and migration of electrons and holes under light excitation and enhance the photoelectric performance of the material. Under the precondition of adding D-(+)-gluconic acid δ-lactone (GDL), BOC/PPy was crosslinked with sodium alginate via Ca^2+^ to obtain a light-programmable BP-SA composite hydrogel. BP-SA hydrogel has strong absorption in the whole band, which provides a good basis for subsequent performance adjustment. When the additive dose of BOC/PPy was 5 wt %, the photosensitivity and photoelectric properties of the hydrogel were the strongest. After five ON/OFF cycles, the hydrogel still had stable photoelectric properties. After irradiation with an 808 nm NIR for 10 min, the temperature of the 5% BP-SA hydrogel increased by 30.2 °C to reach 58.2 °C. The photothermal inhibition rates of 5% BP-SA hydrogel on *S. aureus* and *E. coli* were 99.3% and 99.5%, respectively, and the bacterial surface contracted or was even damaged. After 5 min of white light irradiation with a 500 W xenon lamp, the temperature of the 5% BP-SA hydrogel only increased by 5.2 °C, and there was almost no phototoxicity to the cells. The results show that the BP-SA hydrogel has photoelectric properties under white light irradiation and photothermal properties under NIR irradiation. Under white light irradiation, BP-SA hydrogel is beneficial to somatic cell adhesion and proliferation and promotes angiogenesis. It is speculated that the reason is the formation of electrons and holes in BP-SA to generate electrical signals to induce the increase of Ca^2+^ in human umbilical vein endothelial cells (HUVECs) and to stimulate related downstream signals, followed by modulating HUVECs migration and angiogenesis. In a mouse model of *S. Aureus*-infected wounds on the back, *S. aureus* was reduced by 99.1% after 3 days of BP-SA-NIR treatment. When the wounds of uninfected mice were irradiated with white light for 5 min over an interval of 2 days, the wound area was reduced to 5.8%, with excellent tissue repair. When exposed to NIR light, BP-SA hydrogel is in a photothermal antibacterial state, and when switched to white light, the hydrogel is also switched to a state of promoting wound healing. This study can intelligently combat wound and microbial infection by regulating the light source and effectively treat wounds on demand.

**Table 1:** Summary of conjugated nanomaterials for photothermal agents.

Conjugated Nanomaterials		Examples	Irradiation Conditions	Temperature of the Wound Reached (°C)	PCE	Antimicrobial Activity	Reference
Conjugated small molecules		MCC/CS NPs	606 nm; 500 mW·cm^−2^; 5 min	51.3	66.4%	*E. coli*, MRSA ≈ 100%	[68]
		TCSN (SL, MA, BPA)	1.5 W·cm^−2^; 5 min	50	-	MRSA = 99.99%	[74]
Conjugated oligomers		M2-NPs (BTD introduced with ethynyl groups)	660 nm; 750 mW·cm^−2^; 8 min	52	47.2%	*C. albicans =* 99%; *S. aureus*, *E. coli* = 97%;	[83]
		CP-F8P NPs	808 nm; 1 W·cm^−2^; 5 min	49.5	81.6%	*S. aureus*, *C. albicans* > 99%; Amp^r^ *E. coli* = 99.9%	[84]
Conjugated polymers	PANI	Me-PANI NPs	808 nm; 1.5 W·cm^−2^; 10 min	58	-	*S. aureus*, *E. coli* ≈ 100%	[92]
		PLNP@PANI-GCS	808 nm; 1.5 W·cm^−2^; 5 min	52.2	-	MRSA, *S. aureus*, *E. coli* > 99%	[24]
	PDA	ADPH (agarose/PDA/ε-PL hydrogel)	808 nm; 1 W·cm^−2^; 10 min	59.4	63.9%	*S. aureus*, *E. coli* ≈ 100%	[105]
		B@MPDA-Mal	808 nm; 1 W·cm^−2^; 10 min	44.3	-	MRSA = 95%	[107]
	PPy	PVA-PPy NPs	808 nm; 7 W·cm^−2^; 4 min	-	-	*S. aureus =* 98.3%; *E. coli* = 97.2%	[114]
		BP-SA	808 nm; 10 min	58.2	-	*S. aureus =* 99.3%; *E. coli* = 99.5%	[119]
Pseudo-conjugated polymers		pPCP-NPs	1064 nm; 1 W·cm^−2^; 10 min	52.2	51.5%	ABS-SA, ABS-EC ≈ 100%	[28]

### 2.4. Pseudo-Conjugated Polymers

Conjugated polymers (CPs) have high PCE and excellent photostability, and, in particular, the excitation wavelength of conjugated polymers easily reaches the NIR-II region [53,120]. However, CPs are difficult to degrade in vivo and may have certain biological toxicity, which greatly limits their application in antibacterial and anti-tumor fields. In order to solve this problem, Yu et al. [121] prepared a new type of degradable conjugated polymer by introducing disulfide bonds and named it pseudo-conjugated polymer. Disulfide bonds are easily cleaved by glutathione in vivo, thus achieving degradability for safe and efficient photothermal anti-tumor application. Moreover, Tang et al. [122] prepared a pseudo-conjugated polymer by introducing oxaliplatin, which is easily reduced in the tumor environment to effectively treat cancer through the synergistic treatment of chemotherapy and phototherapy. Pseudo-conjugated polymers not only have the advantages of traditional CPs, but they can also be degraded by active substances in the body, which has great application potential. However, there are few reports about pseudo-conjugated polymers, and in the field of biomedicine, most of them are developed for anti-tumor treatment and few are used for antibacterial applications.

**Figure 12:**
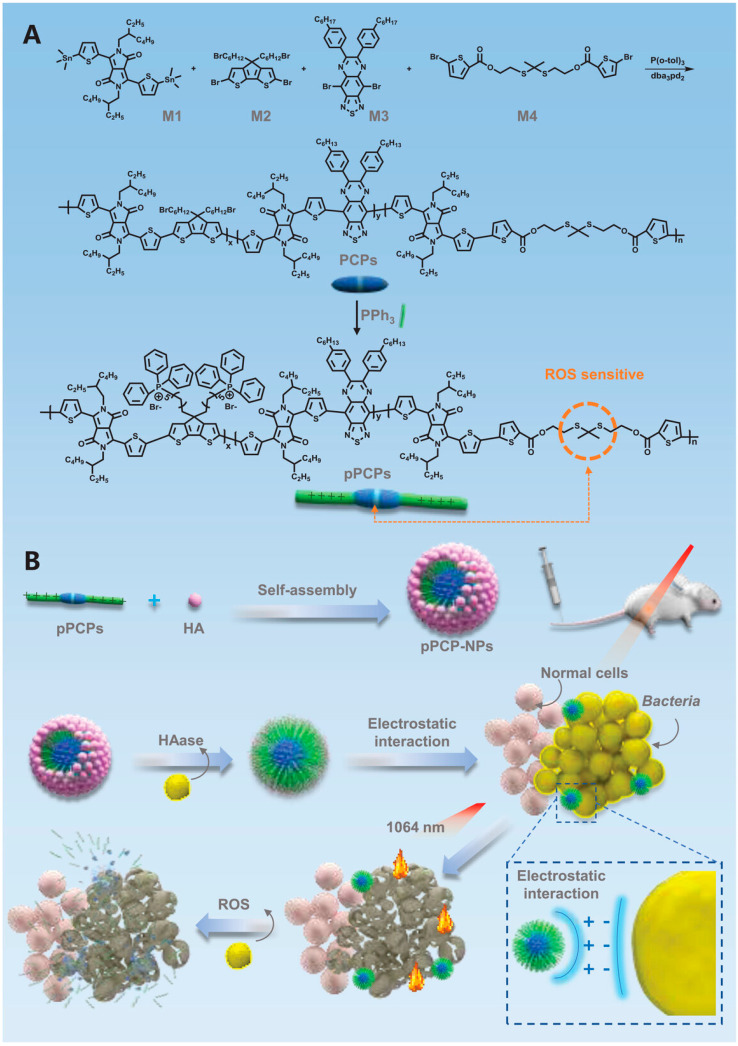
(**A**) The synthesis of pPCPs and (**B**) illustrated design of pPCP-NPs for antibacterial therapy. Reprinted with permission from ref. [28]. Copyright 2022 The Authors.

Zhou et al. [28] designed a ROS-regulated degradating pseudo-conjugated polymer nanoparticle (pPCP-NPs), which can penetrate tissues and target bacteria for efficient NIR-II photothermal antibacterial therapy (Figure 12). The main chain of the photothermal polymer (PCP) containing many sulfur–copper bonds was constructed. In the infected wound area rich in ROS, the sulfur–copper bonds would break and degrade, reducing the biological toxicity. Then, through the Witting reaction, triphenylphosphine (PPh_3_) was introduced to obtain pPCP-containing quaternary phosphorus ion groups. Quaternary phosphorus salt has a larger ionic radius and stronger polarization, so it has a higher affinity for bacteria and improves the antibacterial effect. Then, through electrostatic interaction, the negatively charged hyaluronic acid (HA) was wrapped on the outer crust of the pPCP to form pPCP-NPs with a core–shell structure to reduce the biological toxicity of quaternary phosphorus groups. According to the UV-Vis characterization, pPCP-NPs have a small redshift of 55 nm and two absorption peaks at 734 nm and 1119 nm in an aqueous solution. When 50 μg/mL of the pPCP-NPs solution was irradiated with an NIR-II laser at 1064 nm (1 W·cm^−2^) for 10 min, the temperature was increased to 65 °C, and the photothermal performance remained stable after four ON/OFF cycles. The calculated PCE was 51.5%, which is higher than most of the reported NIR-II materials. When pPCP-NPS was incubated with hyaluronidase (HAase), the HA shell was enzymolized, and the pPCP core was exposed for photothermal antibacterial therapy. Under NIR-II laser irradiation, 50 μg/mL of the pPCP-NPs showed a 100% inhibitory rate against antibiotic-susceptible *S. aureus* (ABS-SA) and *E. coli* (ABS-EC). According to the metabolomics analysis, pPCP-NPs/+L damaged the bacterial DNA, broke the REDOX balance, inhibited carbon/nitrogen utilization and amino acid/nucleotide synthesis, and induced bacterial cell apoptosis. pPCP-NPs coated with a Cy7.5 fluorescent probe were injected intravenously into mice, and fluorescence began to appear after 2 h and lasted for 48 h. After 10 min of laser irradiation at 1064 nm with an intensity of 1 W·cm^−2^, the temperature on the MRSA-infected mice wounds rose to 52.2 °C, which not only can be highly antibacterial but also causes almost no damage to the surrounding healthy tissue. After 8 days of treatment, the MRSA-infected wound was almost completely healed. From the third day of treatment, the weight of the mice gradually increased, and no organ toxicity was observed. This nanoplatform solves the problem that traditional conjugated polymers are not easy to degrade, and provides a new way for antibiotic replacement therapy to be applied.

## 3. Combination Therapy of PTT and PDT Based on Conjugated Nanomaterials

Single PTT requires a high temperature, prolonged laser irradiation, and a high dosage to achieve effective antibacterial activity, which may cause tissue damage and biological toxicity [123]. Therefore, it can be used in combination with other therapies to avoid the above issues and to achieve efficient synergistic antibacterial effects. In PDT, the photosensitizer is stimulated under the irradiation of the light source to produce biotoxic reactive oxygen species (ROS), including ^1^O_2_ and oxygen free radicals, to efficiently kill bacteria [124,125,126]. However, PDT is usually limited by the concentration of the photosensitizer, light flux, and oxygen content. When the threshold is not reached, the ideal antibacterial effect cannot be achieved [127]. Therefore, if PTT and PDT are combined, they can effectively make up for their respective defects.

**Figure 13:**
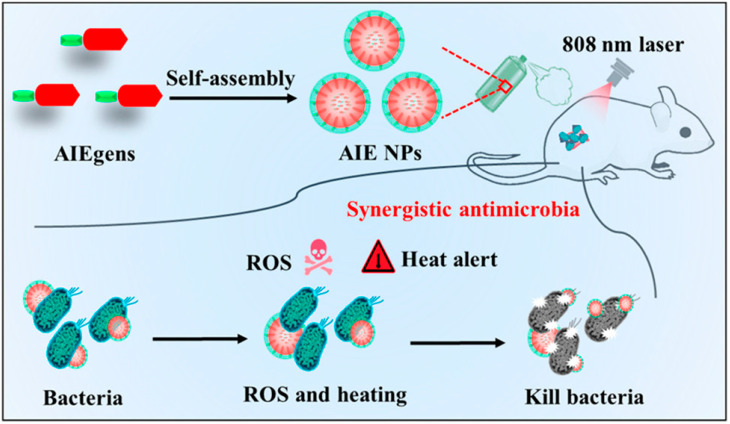
Schematic illustration of AIE-nanoparticle-mediated PTT and PDT for antimicrobial treatment and wound healing. Reprinted with permission from ref. [128]. Copyright 2022 American Chemical Society.

Due to energy dissipation and the complexity of molecular design, integrating all phototherapy modes into a single molecule and achieving accurate spatial co-localization is a challenge. Wang et al. [128] synthesized a conjugated molecule with a highly twisted framework consisting of trianiline, thiophene, and benzo dithiadiazole, for which one end of the main chain was quaternized to provide aggregation-induced luminescence (AIE) properties, followed by assembly into aggregation-induced generation nanoparticles (AIE NPs) in a polar solvent with a hydrated particle size of 72 nm (Figure 13). The surface potential of AIE NPs is 17.2 mV, which is conducive to electrostatically interact with negatively charged pathogenic bacteria. AIE NPs have strong absorption in the NIR region with an emission peak of 685 nm. Under the irradiation of an 808 nm NIR light with an intensity of 0.2 W·cm^−2^, 200 μg/mL of an AIE NPs solution can heat up to 60 °C within 5 min, which is much smaller than the light intensity reported by current photothermal materials. Under the same lighting conditions, it can efficiently produce singlet oxygen with a yield of 72%, which is 18% higher than that of the commercial photosensitizer rose bengal, which is one of the photosensitizers reported to produce singlet oxygen efficiently. Therefore, the newly synthesized AIE NPs have high photothermal conversion ability and photodynamic performance under a single NIR wavelength excitation to realize the unitization of PTT and PDT. AIE NPs can selectively target bacteria, showing significant antibacterial properties against both Gram-positive bacteria (*S. aureus*) and Gram-negative bacteria (*E. coli*). Even at a low light dose, the killing activity of 50 μg/mL of AIE NPs on *S. aureus* and *E. coli* reached 38.7% and 41.5%, respectively. When the light dose was increased to 0.2 W·cm^−2^, 50 μg/mL of AIE NPs killed 99.9% of *S. aureus* and 99.8% of *E. coli*, respectively, suggesting that AIE-NPs-mediated synergistic PTT and PDT are a promising antimicrobial treatment strategy. In addition, AIE NPs have good biocompatibility, showing a 3% hemolysis rate of only in the concentration range of 50–200 μg/mL, and mammalian cells still maintain a 95% survival rate at 100 μg/mL. The particle size and potential of AIE NPs measured after 21 days in PBS are not significantly different from those measured in the first test, which indicates that AIE NPs have good stability under physiological conditions. It was found that after 14 days of wound healing in the group treated with AIE NPs and light (808 nm, 0.2 W·cm^−2^), only 3.4% of the residual wound area was left, while the control group treated without AIE NPs remained at over 80%. This work provides an alternative strategy for the design and preparation of conjugated nanomaterials that activate PTT and PDT functions under the same light source and extend the antimicrobial capabilities of multifunctional conjugated materials.

**Figure 14:**
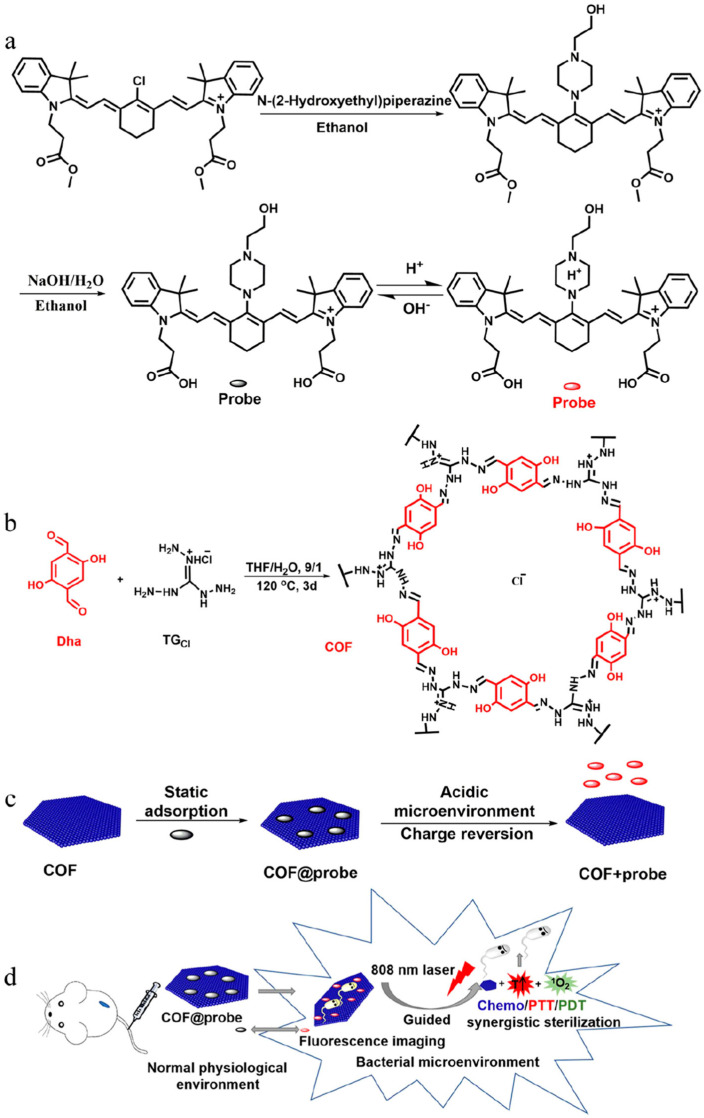
The synthesis of (**a**) probe and (**b**) COF. (**c**) The illustrated action mechanism of COF@probe. (**d**) Illustration of the COF@probe nanoplatform for precise imaging-guided chemo/PTT/PDT synergistic sterilization. Reprinted with permission from ref. [129]. Copyright 2021 American Chemical Society.

Most therapeutic agents are based on the combination of a single photothermal agent and photosensitizer, which are always active and generally lack specificity for bacteria, resulting in uncertain pharmacokinetics and severe non-specific damage to normal tissues. Liu et al. [129] reported a pH-responsive nanoplatform with synergistic chemo-phototherapy capabilities for precise sterilization guided by intelligent fluorescence imaging (Figure 14). A hydroxyethyl piperazine functional cyanine dye was synthesized as the phototherapy agent (probe), and the nitrogen atom on the piperazine ring was used as the specific recognition site of the bacterial acidic microenvironment, which realized intelligent charge reverse orientation and specific sterilization. Introducing carboxyl groups into cyanine molecules increases hydrophilicity and provides self-assembly sites. The combination of a guanidinium-based covalent organic framework (COF) with positive charges and antibacterial activity and smart phototherapy agents is expected to achieve precise and efficient synergistic therapy. Then, the COF@probe platform was prepared by electrostatic self-assembly of the probe and COF. The COF@probe shows the nanosheet structure. The self-assembly of the COF and the probe at a pH of 7.4 (simulating normal physiological pH) makes the zeta potential change from 18.5 ± 0.2 to −26.7 ± 0.4 mV. The positive charge of guanidinium in the COF is balanced with the negative charge of carboxyl in the probe. When the pH varies from 7.4 to 6.0 (simulating the bacterial acidic microenvironment), the zeta potential of the COF@probe quickly reverses to 29.3 ± 0.3 mV due to the protonation of the probe and desorption from the COF. The obtained COF@probe has a characteristic NIR absorption peak of 690 nm at a pH of 7.4, while a distinct redshift peak at 770 nm appears when the pH is adjusted to 6.0, and this process is reversible. At the same time, the intensity and location of the two characteristic absorption peaks are not significantly different from that of the probe with the same concentration, indicating that COF and self-assembly of the probe do not change the intrinsic absorption characteristics of the probe. However, the self-assembled COF@probe causes the obvious fluorescence quenching of the probe at a pH of 7.4, which leads to the quenching of intramolecular and intermolecular fluorescence due to the probe aggregation and π–π accumulation between the probe and the COF, resulting in a low background signal of the COF@probe. However, almost all the adsorbed probes are desorbed from the COF under an acidic environment with good dispersion in the solvent due to the protonation of N atoms in the hydroxyethyl piperazine ring, accompanied by charge reversal, which suppressed the photoinduced electron transfer process, resulting in activated fluorescence at 825 nm. The pKa of the probe measured by fluorescence titration is 6.3 with a pH sensitivity range of 5.5~7.5, which is consistent with the acidic microenvironment of bacteria. Under the irradiation of an 808 nm NIR light (0.6 W·cm^−2^), the COF@probe in an environment with a pH of 6.0 has a temperature change of 12 °C in the 8th minute, while under the same illumination condition, the COF@probe in an environment with a pH of 7.4 has almost no temperature change. Singlet oxygen is produced in the same way as photothermal conversion. In an environment with a pH of 6.0, the COF@probe has obvious singlet oxygen generation, while in an environment with a pH of 7.4, the singlet oxygen signal of the probe is very weak. In vitro antibacterial experiments showed that the probe, COF, and COF@probe caused no obvious damage to *E. coli* or *S. aureus* in the absence of light, but the antibacterial efficiency was close to 100% with the aid of light. Taking the wounds of mice infected with *S. aureus* as the treatment model, a strong fluorescence signal at the infected site could be observed 6 h after injection of the COF@probe into the tail vein, and the fluorescence signal could be maintained in the infected part for 24 h, indicating that the COF@probe has great promise for in vivo bacterial-targeting imaging and the prolongation of blood circulation. The abscess temperature in the treatment group increased rapidly within 10 min of irradiation, and the abscess was gradually relieved and completely healed after 8 days, while the wound area of the control group was still 20–40%, indicating that the PTT/PDT effect of the COF@probe is specifically regulated by the acidic bacterial microenvironment. The wounds of the COF@probe mice in the no-light group also healed to a certain extent, indicating that the COF@probe itself has certain antibacterial abilities and that it plays a role in chemotherapy. The intelligent nanoplatform in this work can not only realize the targeted imaging of bacteria, but it also has the characteristics of chemotherapy/PTT/PDT collaborative efficient sterilization and low side effects, providing it with great practical application potential.

**Figure 15:**
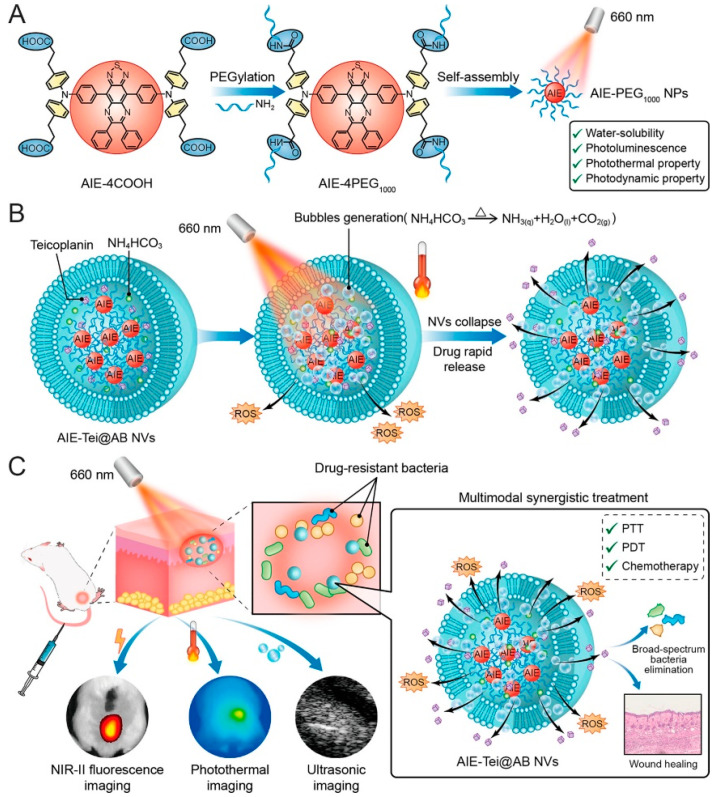
(**A**) The synthesis of AIE-PEG_1000_NPs. (**B**) The illustrated action mechanism of AIE-Tei@AB NVs. (**C**) Schematic illustration of the laser-activated “nanobomb” system for trimodal imaging-guided synergistic PTT-PDT-CDT of drug-resistant bacterial infections. Reprinted with permission from ref. [130]. Copyright 2023 American Chemical Society.

Li et al. [130] designed a 606 nm laser-activated “nanobomb” that has PDT and PTT effects with the release of antibiotics at the same time; it is highly antibacterial under the synergistic action of the three modes of PTT-PDT-CDT (Figure 15). An AIE-4COOH molecule with a D-A-D structure was constructed with 6, 7-diphenyl-[1,2,5] thiadiazole [3, 4-g] quinoline (DPTQ) as the electron acceptor and trianiline containing four carboxyl groups as the electron donor. AIE-4COOH exhibits strong ICT, showing a large stokes shift, and the excitation wavelength is redshifted to the NIR-II region. In the aggregation state, AIE-4COOH’s intramolecular motion is limited, and it can emit bright fluorescence to achieve imaging. Four PEG_1000_ molecules were connected and self-assembled in water to form nanospheres (AIE-PEG_1000_ NPs). The NPs, antibiotic (Tei), and ammonium bicarbonate (AB) were encapsulated in vesicles to obtain AIE-Tei@AB NVs. Under the thermal effect of AIE NPs, AB will decompose to form NH_3_ and CO_2_, a large number of bubbles will cause vesicles to burst, and Tei will be released, achieving antibiotic “shock therapy.” The bubbles show a strong ultrasonic signal, which can realize ultrasonic imaging. After laser irradiation at 606 nm (0.8 W·cm^−2^) for 6 min, 200 μg/mL of the AIE-Tei@AB NVs solution was heated to 63.5 °C. High stability was still maintained after eight ON/OFF cycles with a PCE of 51.5%. AIE-Tei@AB NVs could eliminate 99.9% of MRSA in vitro. When the drug was added to the abscess areas of mice, the wound temperature rose to 53 °C within 5 min of illumination, and the clearance rate of MRSA reached 99.1%. The “nanobomb” multi-modal antimicrobial strategy provides good insight for the treatment of microbial infection.

## 4. Summary and Prospects

Compared with traditional treatment methods, PTT is not prone to developing drug resistance and can treat microbial infections non-invasively and efficiently, which has made it an attractive therapy. The selection of PTAs is particularly important in PTT. Compared with inorganic PTAs with high biotoxicity, organic conjugated nanomaterials have high biocompatibility, easy modification, and good photothermal properties with great potential in PTT. According to their molecular weight and structure, conjugated materials can be divided into conjugated small molecules, conjugated oligomers, conjugated polymers, and pseudo-conjugated molecules. The PCE of conjugated small molecules and conjugated oligomers can be improved by designing the molecular structure, and the redshift of the absorption peak can be achieved. The general strategy is to introduce a D-A structure, enhance ICT, reduce ΔEst, and then promote the energy dissipation of excited states through non-radiative ways to improve the photothermal performance. Branch chains can also be introduced to improve the light absorption capacity of PTA. Common conjugated polymers include PANI, PDA, and PPy. However, their water solubility is usually poor, so in the preparation of PTAs, it is necessary to add surfactants or to introduce hydrophilic substances, such as PEG, to improve the solubility of conjugated polymers. Conjugated polymers are easy to aggregate and reduce the photothermal conversion ability, which can be solved by uniformly dispersing in hydrogels to improve PCE. However, conjugated polymers are difficult to biodegrade and may potentially be toxic. Pseudo-conjugated polymers solve this problem cleverly, and they can achieve degradation under the specific environment triggered by a microbial infection in organisms. At the same time, they also have NIR-II response characteristics and good photothermal properties, so they have great potential for biological application. A single PTT may cause irreversible thermal damage to the surrounding healthy tissue due to a high temperature. By combining with PDT, excellent antibacterial effects can be achieved under mild conditions.

Although PTT based on conjugated materials has made more achievements in combating microbial infection, there are still some problems to be solved. The first is biosafety: if PTAs cannot be degraded in time, long-term accumulation in the body may cause certain harm to the body. The second is targeting, as the temperature often rises to more than 60 °C in the treatment process, and if the conjugated nanomaterials cannot accurately locate the infection site, it is easy to produce “accidental injury” to the healthy tissue, making the problem more difficult. Finally, real-time monitoring: microorganisms have a strong proliferation ability, and if pathogens are not completely eradicated, they may continue to proliferate and cause secondary infection. Therefore, real-time monitoring and on-demand drug delivery are required. In the future, real-time monitoring, rapid targeting, biosecurity, and multi-mode therapy are the development trends of PTT based on conjugated nanomaterials. We hope that this review can give readers a certain understanding of PTT based on conjugated materials and provide some ideas for related research.

## Data Availability

Data sharing not applicable.

## References

[1] Mehrabi M.R., Soltani M., Chiani M., Raahemifar K., Farhangi A. (2023). Nanomedicine: New Frontiers in Fighting Microbial Infections. Nanomaterials.

[2] McCulloch T.R., Wells T.J., Souza-Fonseca-Guimaraes F. (2022). Towards efficient immunotherapy for bacterial infection. Trends Microbiol..

[3] Baran A., Kwiatkowska A., Potocki L. (2023). Antibiotics and Bacterial Resistance-A Short Story of an Endless Arms Race. Int. J. Mol. Sci..

[4] Munir M.U., Ahmad M.M. (2022). Nanomaterials Aiming to Tackle Antibiotic-Resistant Bacteria. Pharmaceutics.

[5] Idris F.N., Nadzir M.M. (2023). Multi-drug resistant ESKAPE pathogens and the uses of plants as their antimicrobial agents. Arch. Microbiol..

[6] Gajdacs M., Albericio F. (2019). Antibiotic Resistance: From the Bench to Patients. Antibiotics.

[7] Bai X., Yang Y., Zheng W., Huang Y., Xu F., Bao Z. (2023). Synergistic photothermal antibacterial therapy enabled by multifunctional nanomaterials: Progress and perspectives. Mater. Chem. Front..

[8] Pulingam T., Parumasivam T., Gazzali A.M., Sulaiman A.M., Chee J.Y., Lakshmanan M., Chin C.F., Sudesh K. (2022). Antimicrobial resistance: Prevalence, economic burden, mechanisms of resistance and strategies to overcome. Eur. J. Pharm. Sci..

[9] Roemhild R., Bollenbach T., Andersson D.I. (2022). The physiology and genetics of bacterial responses to antibiotic combinations. Nat. Rev. Microbiol..

[10] Nichols R.J., Sen S., Choo Y.J., Beltrao P., Zietek M., Chaba R., Lee S., Kazmierczak K.M., Lee K.J., Wong A. (2011). Phenotypic landscape of a bacterial cell. Cell.

[11] Minato Y., Dawadi S., Kordus S.L., Sivanandam A., Aldrich C.C., Baughn A.D. (2018). Mutual potentiation drives synergy between trimethoprim and sulfamethoxazole. Nat. Commun..

[12] Songca S.P., Adjei Y. (2022). Applications of Antimicrobial Photodynamic Therapy against Bacterial Biofilms. Int. J. Mol. Sci..

[13] Yougbare S., Chou H.L., Yang C.H., Krisnawati D.I., Jazidie A., Nuh M., Kuo T.R. (2021). Facet-dependent gold nanocrystals for effective photothermal killing of bacteria. J. Hazard. Mater..

[14] Cheng S., Chen L., Gong F., Yang X., Han Z., Wang Y., Ge J., Gao X., Li Y., Zhong X. (2023). PtCu Nanosonosensitizers with Inflammatory Microenvironment Regulation for Enhanced Sonodynamic Bacterial Elimination and Tissue Repair. Adv. Funct. Mater..

[15] Chen L., Xing S., Lei Y., Chen Q., Zou Z., Quan K., Qing Z., Liu J., Yang R. (2021). A Glucose-Powered Activatable Nanozyme Breaking pH and H(2) O(2) Limitations for Treating Diabetic Infections. Angew. Chem. Int. Ed..

[16] Wang X.W., Shi Q.Q., Zha Z.B., Zhu D.D., Zheng L.R., Shi L.X., Wei X.W., Lian L., Wu K.L., Cheng L. (2021). Copper single-atom catalysts with photothermal performance and enhanced nanozyme activity for bacteria-infected wound therapy. Bioact. Mater..

[17] Yu C.H., Sui S.Y., Yu X.T., Huang W.L., Wu Y.F., Zeng X., Chen Q.M., Wang J., Peng Q. (2022). Ti3C2Tx MXene loaded with indocyanine green for synergistic photothermal and photodynamic therapy for drug-resistant bacterium. Colloids Surf. B-Biointerfaces.

[18] Liu X., Yang Y.Y., Ling M.J., Sun R., Zhu M.Y., Chen J.J., Yu M., Peng Z.W., Yu Z.Q., Liu X.Q. (2021). Near-Infrared II Light-Triggered Robust Carbon Radical Generation for Combined Photothermal and Thermodynamic Therapy of Hypoxic Tumors. Adv. Funct. Mater..

[19] Yang X., Li H.P., Qian C.G., Guo Y.X., Li C.Z., Gao F., Yang Y., Wang K.K., Oupicky D., Sun M.J. (2018). Near-infrared light-activated IR780-loaded liposomes for anti-tumor angiogenesis and Photothermal therapy. Nanomed. Nanotechnol. Biol. Med..

[20] He W., Wang Z.Y., Bai H.T., Zhao Z., Kwok R.T.K., Lam J.W.Y., Tang B.Z. (2021). Highly efficient photothermal nanoparticles for the rapid eradication of bacterial biofilms. Nanoscale.

[21] Shanmugam V., Selvakumar S., Yeh C.S. (2014). Near-infrared light-responsive nanomaterials in cancer therapeutics. Chem. Soc. Rev..

[22] Korupalli C., Kalluru P., Nuthalapati K., Kuthala N., Thangudu S., Vankayala R. (2020). Recent Advances of Polyaniline-Based Biomaterials for Phototherapeutic Treatments of Tumors and Bacterial Infections. Bioengineering.

[23] Li L., Han X., Wang M., Li C., Jia T., Zhao X. (2021). Recent advances in the development of near-infrared organic photothermal agents. Chem. Eng. J..

[24] Yan L.X., Chen L.J., Zhao X., Yan X.P. (2020). pH Switchable Nanoplatform for In Vivo Persistent Luminescence Imaging and Precise Photothermal Therapy of Bacterial Infection. Adv. Funct. Mater..

[25] Lapotko D.O. (2013). Nanophotonics and Theranostics: Will Light Do the Magic?. Theranostics.

[26] Qin Z.J., Ren T.B., Zhou H.J., Zhang X.X., He L., Li Z., Zhang X.B., Yuan L. (2022). NIRII-HDs: A Versatile Platform for Developing Activatable NIR-II Fluorogenic Probes for Reliable In Vivo Analyte Sensing. Angew. Chem. Int. Ed..

[27] Wu M., Huang Y.X., Huang X.Y., Wang F., Wei X.B. (2023). Copolymerized carbon nitride nanoparticles for near-infrared II photoacoustic-guided synergistic photothermal/radiotherapy. Front. Chem..

[28] Zhou H., Tang D., Kang X., Yuan H., Yu Y., Xiong X., Wu N., Chen F., Wang X., Xiao H. (2022). Degradable Pseudo Conjugated Polymer Nanoparticles with NIR-II Photothermal Effect and Cationic Quaternary Phosphonium Structural Bacteriostasis for Anti-Infection Therapy. Adv. Sci..

[29] Jiang Y., Li J., Zhen X., Xie C., Pu K. (2018). Dual-Peak Absorbing Semiconducting Copolymer Nanoparticles for First and Second Near-Infrared Window Photothermal Therapy: A Comparative Study. Adv. Mater..

[30] Zhang Y., Li Y., Li J.Y., Mu F., Wang J., Shen C., Wang H., Huang F., Chen B., Luo Z.M. (2023). DNA-Templated Ag@Pd Nanoclusters for NIR-II Photoacoustic Imaging-Guided Photothermal-Augmented Nanocatalytic Therapy. Adv. Healthc. Mater..

[31] Shi W.Q., Han Q.R., Wu J.J., Ji C.Y., Zhou Y.Q., Li S.H., Gao L.P., Leblanc R.M., Peng Z.L. (2022). Synthesis Mechanisms, Structural Models, and Photothermal Therapy Applications of Top-Down Carbon Dots from Carbon Powder, Graphite, Graphene, and Carbon Nanotubes. Int. J. Mol. Sci..

[32] Qie X.W., Zan M.H., Gui P., Chen H.Y., Wang J.K., Lin K.C., Mei Q., Ge M.F., Zhang Z.Q., Tang Y.G. (2022). Design, Synthesis, and Application of Carbon Dots With Synergistic Antibacterial Activity. Front. Bioeng. Biotechnol..

[33] Guan M., Chu G., Jin J., Liu C., Cheng L., Guo Y., Deng Z., Wang Y. (2022). A Combined Cyanine/Carbomer Gel Enhanced Photodynamic Antimicrobial Activity and Wound Healing. Nanomaterials.

[34] Shi J.P., Li J., Wang Y., Cheng J.J., Zhang C.Y. (2020). Recent advances in MoS_2_-based photothermal therapy for cancer and infectious disease treatment. J. Mater. Chem. B.

[35] Zhang W.W., Kuang Z., Song P., Li W.Z., Gui L., Tang C.C., Tao Y.G., Ge F., Zhu L.B. (2022). Synthesis of a Two-Dimensional Molybdenum Disulfide Nanosheet and Ultrasensitive Trapping of Staphylococcus Aureus for Enhanced Photothermal and Antibacterial Wound-Healing Therapy. Nanomaterials.

[36] Li C.N., Lin W.H., Liu S., Sun T.T., Xie Z.G. (2021). Structural optimization of organic fluorophores for highly efficient photothermal therapy. Mater. Chem. Front..

[37] Song X.J., Chen Q., Liu Z. (2015). Recent advances in the development of organic photothermal nano-agents. Nano Res..

[38] Liu L.C., Wang W.F., Hong W.H., Jin Y.Y., Wang L.C., Liu S.J., Wang A.L., Liu X.S. (2022). Photothermal 2D Nanosheets Combined with Astragaloside IV for Antibacterial Properties and Promoting Angiogenesis to Treat Infected Wounds. Front. Bioeng. Biotechnol..

[39] Liu C., Zhang S., Li J., Wei J., Muellen K., Yin M. (2019). A Water-Soluble, NIR-Absorbing Quaterrylenediimide Chromophore for Photoacoustic Imaging and Efficient Photothermal Cancer Therapy. Angew. Chem. Int. Ed..

[40] Liu T., Zhang M., Liu W., Zeng X., Song X., Yang X., Zhang X., Feng J. (2018). Metal Ion/Tannic Acid Assembly as a Versatile Photothermal Platform in Engineering Multimodal Nanotheranostics for Advanced Applications. ACS Nano.

[41] Chen Z., Li J., Zhou J., Chen X. (2023). Photothermal Janus PPy-SiO_2_@PAN/F-SiO_2_@PVDF-HFP membrane for high-efficient, low energy and stable desalination through solar membrane distillation. Chem. Eng. J..

[42] Sarkar S., Levi-Polyachenko N. (2020). Conjugated polymer nano-systems for hyperthermia, imaging and drug delivery. Adv. Drug Deliv. Rev..

[43] Zhang Y.G., Zhang S.Y., Zhang Z.H., Ji L.L., Zhang J.M., Wang Q.H., Guo T., Ni S.M., Cai R., Mu X.Y. (2021). Recent Progress on NIR-II Photothermal Therapy. Front. Chem..

[44] Liu Y., Li F., Guo Z., Xiao Y., Zhang Y., Sun X., Zhe T., Cao Y., Wang L., Lu Q. (2020). Silver nanoparticle-embedded hydrogel as a photothermal platform for combating bacterial infections. Chem. Eng. J..

[45] Jung H.S., Verwilst P., Sharma A., Shin J., Sessler J.L., Kim J.S. (2018). Organic molecule-based photothermal agents: An expanding photothermal therapy universe. Chem. Soc. Rev..

[46] Wei D., Yu Y., Huang Y., Jiang Y., Zhao Y., Nie Z., Wang F., Ma W., Yu Z., Huang Y. (2021). A Near-Infrared-II Polymer with Tandem Fluorophores Demonstrates Superior Biodegradability for Simultaneous Drug Tracking and Treatment Efficacy Feedback. ACS Nano.

[47] Wang Y., Zhang H., Wang Z., Feng L. (2020). Photothermal Conjugated Polymers and Their Biological Applications in Imaging and Therapy. ACS Appl. Polym. Mater..

[48] Qi J., Ou H., Liu Q., Ding D. (2021). Gathering brings strength: How organic aggregates boost disease phototheranostics. Aggregate.

[49] Lin F., Duan Q.-Y., Wu F.-G. (2020). Conjugated Polymer-Based Photothermal Therapy for Killing Microorganisms. ACS Appl. Polym. Mater..

[50] Xu L., Cheng L., Wang C., Peng R., Liu Z. (2014). Conjugated polymers for photothermal therapy of cancer. Polym. Chem..

[51] Ponzio R.A., Ibarra L.E., Achilli E.E., Odella E., Chesta C.A., Martinez S.R., Palacios R.E. (2022). Sweet light o? mine: Photothermal and photodynamic inactivation of tenacious pathogens using conjugated polymers. J. Photochem. Photobiol. B-Biol..

[52] Lu B., Huang Y.Y., Zhang Z.C., Quan H., Yao Y. (2022). Organic conjugated small molecules with donor-acceptor structures: Design and application in the phototherapy of tumors. Mater. Chem. Front..

[53] Wang Y., Meng H.-M., Song G., Li Z., Zhang X.-B. (2020). Conjugated-Polymer-Based Nanomaterials for Photothermal Therapy. ACS Appl. Polym. Mater..

[54] Geng J., Sun C., Liu J., Liao L.-D., Yuan Y., Thakor N., Wang J., Liu B. (2015). Biocompatible Conjugated Polymer Nanoparticles for Efficient Photothermal Tumor Therapy. Small.

[55] Ito T., Shirakawa H., Ikeda S. (1996). Simultaneous polymerization and formation of polyacetylene film on the surface of concentrated soluble Ziegler-type catalyst solution. J. Polym. Sci. Part A Polym. Chem..

[56] Jia W., Huang F., Zhang Q., Zhao L., Li C., Lu Y. (2022). Novel conjugated small molecule-based nanoparticles for NIR-II photothermal antibacterial therapy. Chem. Commun..

[57] Chen J., Wen K., Chen H., Jiang S., Wu X., Lv L., Peng A., Zhang S., Huang H. (2020). Achieving High-Performance Photothermal and Photodynamic Effects upon Combining D-A Structure and Nonplanar Conformation. Small.

[58] Wu B., Fu J., Zhou Y., Shi Y., Wang J., Feng X., Zhao Y., Zhou G., Lu C., Quan G. (2019). Metal-Organic Framework-Based Chemo-Photothermal Combinational System for Precise, Rapid, and Efficient Antibacterial Therapeutics. Pharmaceutics.

[59] Ting C.-W., Chou Y.-H., Huang S.-Y., Chiang W.-H. (2021). Indocyanine green-carrying polymeric nanoparticles with acid-triggered detachable PEG coating and drug release for boosting cancer photothermal therapy. Colloids Surf. B-Biointerfaces.

[60] Sahu A., Choi W.I., Lee J.H., Tae G. (2013). Graphene oxide mediated delivery of methylene blue for combined photodynamic and photothermal therapy. Biomaterials.

[61] Lv Y., Li P., Su R., Cai J., Zhong H., Wen F., Su W. (2023). Methylene Blue/Carbon Dots Composite with Photothermal and Photodynamic Properties: Synthesis, Characterization, and Antibacterial Application. Photochem. Photobiol..

[62] Ding Y., Wang C., Lu B., Yao Y. (2021). Enhancing the Stability and Photothermal Conversion Efficiency of ICG by Pillar 5 arene-Based Host-Guest Interaction. Front. Chem..

[63] Lu B., Zhang Z., Jin D., Yuan X., Wang J., Ding Y., Wang Y., Yao Y. (2021). A-DA′D-A fused-ring small molecule-based nanoparticles for combined photothermal and photodynamic therapy of cancer. Chem. Commun..

[64] Zhao Y., Dai W., Peng Y., Niu Z., Sun Q., Shan C., Yang H., Verma G., Wojtas L., Yuan D. (2020). A Corrole-Based Covalent Organic Framework Featuring Desymmetrized Topology. Angew. Chem. Int. Ed..

[65] You L., Shen H., Shi L., Zhang G., Liu H., Wang H., Ji L. (2010). Photophysical properties of the Corrole photosensitizers. Sci. China-Phys. Mech. Astron..

[66] Jiang X., Liu R.-X., Liu H.-Y., Chang C.K. (2019). Corrole-based photodynamic antitumor therapy. J. Chin. Chem. Soc..

[67] Shao W., Wang H., He S., Shi L., Peng K., Lin Y., Zhang L., Ji L., Liu H. (2012). Photophysical Properties and Singlet Oxygen Generation of Three Sets of Halogenated Corroles. J. Phys. Chem. B.

[68] Yu Y., Tian R., Zhao Y., Qin X., Hu L., Zou J.-J., Yang Y.-W., Tian J. (2022). Self-Assembled Corrole/Chitosan Photothermal Nanoparticles for Accelerating Infected Diabetic Wound Healing. Adv. Healthc. Mater..

[69] Liu G., Wang Z., Sun W., Lin X., Wang R., Li C., Zong L., Fu Z., Liu H., Xu S. (2023). Robust emission in near-infrared II of lanthanide nanoprobes conjugated with Au (LNPs-Au) for temperature sensing and controlled photothermal therapy. Chem. Eng. J..

[70] Wu X., Zhao G., Ruan Y., Feng K., Gao M., Liu Y., Sun X. (2023). Temperature-Responsive Nanoassemblies for Self-Regulated Photothermal Therapy and Controlled Copper Release to Accelerate Chronic Wound Healing. ACS Appl. Bio Mater..

[71] Xia Y., Li C., Cao J., Chen Z., Wang J., Wu Y., Zhang X. (2022). Liposome-templated gold nanoparticles for precisely temperature-controlled photothermal therapy based on heat shock protein expression. Colloids Surf. B-Biointerfaces.

[72] Shen S., Feng L., Qi S., Cao J., Ge Y., Wu L., Wang S. (2020). Reversible Thermochromic Nanoparticles Composed of a Eutectic Mixture for Temperature-Controlled Photothermal Therapy. Nano Lett..

[73] Xu D., He X., Obeng E., Ye Z., Shen J., Ding X. (2022). Two-dimensional NbS2 nanosheets with hyperthermia for killing bacteria to promote infected wound healing. Mater. Des..

[74] Wang J.X., Hao B.Y., Xue K., Fu H., Xiao M.H., Zhang Y.X., Shi L.Q., Zhu C.L. (2022). A Smart Photothermal Nanosystem with an Intrinsic Temperature-Control Mechanism for Thermostatic Treatment of Bacterial Infections. Adv. Mater..

[75] Shao W., Wei Q., Wang S., Li F., Wu J., Ren J., Cao F., Liao H., Gao J.-Q., Zhou M. (2020). Molecular engineering of D-A-D conjugated small molecule nanoparticles for high performance NIR-II photothermal therapy. Mater. Horiz..

[76] Liu S., Zhou X., Zhang H., Ou H., Lam J.W.Y., Liu Y., Shi L., Ding D., Tang B.Z. (2019). Molecular Motion in Aggregates: Manipulating TICT for Boosting Photothermal Theranostics. J. Am. Chem. Soc..

[77] Wu Q., Zhu Y., Fang X., Hao X., Jiao L., Hao E., Zhang W. (2020). Conjugated BODIPY Oligomers with Controllable Near-Infrared Absorptions as Promising Phototheranostic Agents through Excited-State Intramolecular Rotations. ACS Appl. Mater. Interfaces.

[78] Jia S., Li Z., Shao J., Yuan H., Li L., Xu L. (2022). Acceptor Regulation of Acceptor-Donor-Acceptor Type Conjugated Oligomer for Photothermal Combating of Resistant Bacteria. ACS Appl. Polym. Mater..

[79] Zhu C.L., Yang Q.O., Liu L.B., Lv F.T., Li S.Y., Yang G.Q., Wang S. (2011). Multifunctional Cationic Poly(p-phenylene vinylene) Polyelectrolytes for Selective Recognition, Imaging, and Killing of Bacteria Over Mammalian Cells. Adv. Mater..

[80] Zhou L., Lv F., Liu L., Wang S. (2019). Water-Soluble Conjugated Organic Molecules as Optical and Electrochemical Materials for Interdisciplinary Biological Applications. Acc. Chem. Res..

[81] He Y., Li N., Yang S., Tan X., Tang L., Yang Q. (2023). Near-Infrared Molecular Photosensitizer Decorated with Quaternary Ammonium for High-Efficiency Photothermal Treatment of Bacterial Infections. Chemosensors.

[82] Li X., Liu L., Li S., Wan Y., Chen J.-X., Tian S., Huang Z., Xiao Y.-F., Cui X., Xiang C. (2019). Biodegradable pi-Conjugated Oligomer Nanoparticles with High Photothermal Conversion Efficiency for Cancer Theranostics. ACS Nano.

[83] Zhang H., Guo L., Wang Y., Feng L. (2021). Molecular engineering to boost the photothermal effect of conjugated oligomer nanoparticles. Biomater. Sci..

[84] Yuan H., Li Z., Zhao Q., Jia S., Wang T., Xu L., Yuan H., Li S. (2023). Molecular Evolution of Acceptor-Donor-Acceptor-type Conjugated Oligomer Nanoparticles for Efficient Photothermal Antimicrobial Therapy. Adv. Funct. Mater..

[85] Della Pina C., Falletta E. (2022). Advances in Polyaniline for Biomedical Applications. Curr. Med. Chem..

[86] Hong Y., Cho W., Kim J., Hwng S., Lee E., Heo D., Ku M., Suh J.S., Yang J., Kim J.H. (2016). Photothermal ablation of cancer cells using self-doped polyaniline nanoparticles. Nanotechnology.

[87] Wang J.P., Yan R., Guo F., Yu M., Tan F.P., Li N. (2016). Targeted lipid-polyaniline hybrid nanoparticles for photoacoustic imaging guided photothermal therapy of cancer. Nanotechnology.

[88] Zhou J., Lu Z.G., Zhu X.J., Wang X.J., Liao Y., Ma Z.F., Li F.Y. (2013). NIR photothermal therapy using polyaniline nanoparticles. Biomaterials.

[89] Bidez P.R., Li S.X., MacDiarmid A.G., Venancio E.C., Wei Y., Lelkes P.I. (2006). Polyaniline, an electroactive polymer, supports adhesion and proliferation of cardiac myoblasts. J. Biomater. Sci. Polym. Ed..

[90] Ghahremanloo A., Zare E.N., Salimi F., Makvandi P. (2022). Electroconductive and photoactive poly(phenylenediamine)s with antioxidant and antimicrobial activities for potential photothermal therapy. New J. Chem..

[91] Yang J., Choi J., Bang D., Kim E., Lim E.K., Park H., Suh J.S., Lee K., Yoo K.H., Kim E.K. (2011). Convertible Organic Nanoparticles for Near-Infrared Photothermal Ablation of Cancer Cells. Angew. Chem. Int. Ed..

[92] Pang Q., Wu K.H., Jiang Z.L., Shi Z.W., Si Z.Z., Wang Q., Cao Y.H., Hou R.X., Zhu Y.B. (2022). A Polyaniline Nanoparticles Crosslinked Hydrogel with Excellent Photothermal Antibacterial and Mechanical Properties for Wound Dressing. Macromol. Biosci..

[93] Ruppel S.S., Liang J. (2022). Tunable Properties of Polydopamine Nanoparticles and Coated Surfaces. Langmuir.

[94] Bi Z., Teng H., Li Q., Zhang S. (2022). Enhanced Carboxymethylcellulose Sponge for Hemostasis and Wound Repair. Front. Mater..

[95] Barclay T.G., Hegab H.M., Clarke S.R., Ginic-Markovic M. (2017). Versatile Surface Modification Using Polydopamine and Related Polycatecholamines: Chemistry, Structure, and Applications. Adv. Mater. Interfaces.

[96] Liu Y., Ai K., Lu L. (2014). Polydopamine and Its Derivative Materials: Synthesis and Promising Applications in Energy, Environmental, and Biomedical Fields. Chem. Rev..

[97] Chinchulkar S.A., Patra P., Dehariya D., Yu A., Rengan A.K. (2022). Polydopamine nanocomposites and their biomedical applications: A review. Polym. Adv. Technol..

[98] Alkan-Tas B., Berksun E., Tas C.E., Unal S., Unal H. (2022). NIR-responsive waterborne polyurethane-polydopamine coatings for light-driven disinfection of surfaces. Prog. Org. Coat..

[99] Wang C., Bai J., Liu Y., Jia X., Jiang X. (2016). Polydopamine Coated Selenide Molybdenum: A New Photothermal Nanocarrier for Highly Effective Chemo-Photothermal Synergistic Therapy. ACS Biomater. Sci. Eng..

[100] Cao H., Jiang B., Yang Y., Zhao M., Sun N., Xia J., Gao X., Li J. (2021). Cell membrane covered polydopamine nanoparticles with two-photon absorption for precise photothermal therapy of cancer. J. Colloid Interface Sci..

[101] Fan S., Lin W., Huang Y., Xia J., Xu J.-F., Zhang J., Pi J. (2022). Advances and Potentials of Polydopamine Nanosystem in Photothermal-Based Antibacterial Infection Therapies. Front. Pharmacol..

[102] Zou Y., Chen X.F., Yang P., Liang G.J., Yang Y., Gu Z.P., Li Y.W. (2020). Regulating the absorption spectrum of polydopamine. Sci. Adv..

[103] Qi X., Huang Y., You S., Xiang Y., Cai E., Mao R., Pan W., Tong X., Dong W., Ye F. (2022). Engineering Robust Ag-Decorated Polydopamine Nano-Photothermal Platforms to Combat Bacterial Infection and Prompt Wound Healing. Adv. Sci..

[104] Li Z., You S., Mao R., Xiang Y., Cai E., Deng H., Shen J., Qi X. (2022). Architecting polyelectrolyte hydrogels with Cu-assisted polydopamine nanoparticles for photothermal antibacterial therapy. Mater. Today Bio.

[105] Qi X., Pan W., Tong X., Gao T., Xiang Y., You S., Mao R., Chi J., Hu R., Zhang W. (2021). ε-Polylysine-stabilized agarose/polydopamine hydrogel dressings with robust photothermal property for wound healing. Carbohydr. Polym..

[106] Mohamed Salleh N.A.b., Tanaka Y., Sutarlie L., Su X. (2022). Detecting bacterial infections in wounds: A review of biosensors and wearable sensors in comparison with conventional laboratory methods. Analyst.

[107] Lv K., Li G., Pan X., Liu L., Chen Z., Zhang Y., Xu H., Ma D. (2023). Bacteria-Targeted Combined with Photothermal/NO Nanoparticles for the Treatment and Diagnosis of MRSA Infection In Vivo. Adv. Healthc. Mater..

[108] Tan Y., Ghandi K. (2013). Kinetics and mechanism of pyrrole chemical polymerization. Synth. Met..

[109] Kang H., Lee W., Oh J., Kim T., Lee C., Kim B.J. (2016). From Fullerene-Polymer to All-Polymer Solar Cells: The Importance of Molecular Packing, Orientation, and Morphology Control. Acc. Chem. Res..

[110] Fan Y.Z., Wang Z.A., Ren W.Z., Liu G.Y., Xing J., Xiao T.Z., Li W., Li Y.J., Yu P., Ning C.Y. (2022). Space-Confined Synthesis of Thin Polypyrrole Nanosheets in Layered Bismuth Oxychloride for a Photoresponse Antibacterial within the Near-Infrared Window and Accelerated Wound Healing. ACS Appl. Mater. Interfaces.

[111] Wang M.Z. (2016). Emerging Multifunctional NIR Photothermal Therapy Systems Based on Polypyrrole Nanoparticles. Polymers.

[112] Alizadeh N., Akbarinejad A., Hosseinkhani S., Rabbani F. (2019). Synthesis of highly fluorescent water-soluble polypyrrole for cell imaging and iodide ion sensing. Anal. Chim. Acta.

[113] Antony M.J., Jayakannan M. (2007). Amphiphilic azobenzenesulfonic acid anionic surfactant for water-soluble, ordered, and luminescent polypyrrole nanospheres. J. Phys. Chem. B.

[114] Wang Y., He X.D., Cheng Y.F., Li L., Zhang K., Kang E.T., Xu L.Q. (2022). Surface co-deposition of polypyrrole nanoparticles and tannic acid for photothermal bacterial eradication. Colloids Surf. B-Biointerfaces.

[115] Tang Q.W., Ke Q., Chen Q., Zhang X.Y., Su J.Y., Ning C.Y., Fang L.M. (2023). Flexible, Breathable, and Self-Powered Patch Assembled of Electrospun Polymer Triboelectric Layers and Polypyrrole-Coated Electrode for Infected Chronic Wound Healing. ACS Appl. Mater. Interfaces.

[116] Luo R.Z., Dai J.Y., Zhang J.P., Li Z. (2021). Accelerated Skin Wound Healing by Electrical Stimulation. Adv. Healthc. Mater..

[117] Weng Z.Z., Yu F., Leng Q.H., Zhao S.Y., Xu Y.Y., Zhang W., Zhu Z.L., Ye J., Wei Q., Wang X.L. (2021). Electrical and visible light dual-responsive ZnO nanocomposite with multiple wound healing capability. Mater. Sci. Eng. C-Mater. Biol. Appl..

[118] Qiao Z., Ding J., Wu C.H., Zhou T., Wu K., Zhang Y.S., Xiao Z.W., Wei D., Sun J., Fan H.S. (2023). One-Pot Synthesis of Bi_2_S_3_/TiO_2_/rGO Heterostructure with Red Light-Driven Photovoltaic Effect for Remote Electrotherapy-Assisted Wound Repair. Small.

[119] Zhang Z.K., Xie J.N., Xing J., Li C.H., Wong T.M., Yu H., Li Y.X., Yang F.B., Tian Y., Zhang H. (2023). Light-Programmable Nanocomposite Hydrogel for State-Switchable Wound Healing Promotion and Bacterial Infection Elimination. Adv. Healthc. Mater..

[120] He Y.L., Cao Y.Y., Wang Y.P. (2018). Progress on Photothermal Conversion in the Second NIR Window Based on Conjugated Polymers. Asian J. Org. Chem..

[121] Yu Y., Tang D., Liu C., Zhang Q., Tang L., Lu Y., Xiao H. (2022). Biodegradable Polymer with Effective Near-Infrared-II Absorption as a Photothermal Agent for Deep Tumor Therapy. Adv. Mater..

[122] Tang D., Zhou H., Cui M., Liang G., Zhang H., Xiao H. (2023). NIR-II Light Accelerated Prodrug Reduction of Pt(IV)-Incorporating Pseudo Semiconducting Polymers for Robust Degradation and Maximized Photothermal/Chemo-Immunotherapy. Adv. Mater..

[123] Naskar A., Kim K.S. (2023). Friends against the Foe: Synergistic Photothermal and Photodynamic Therapy against Bacterial Infections. Pharmaceutics.

[124] Hu X.J., Zhang H., Wang Y.T., Shiu B.C., Lin J.H., Zhang S.J., Lou C.W., Li T.T. (2022). Synergistic antibacterial strategy based on photodynamic therapy: Progress and perspectives. Chem. Eng. J..

[125] Yin C., Wang Z., Dai C., Yang B., Wang W., Yang E., Guo F., Fan C., Zhang P., Sun J. (2023). Light-triggered photosynthetic engineered bacteria for enhanced-photodynamic therapy by relieving tumor hypoxic microenvironment. Theranostics.

[126] Wang D., Li H.J., Ji L., Liu J., Li Y., Xu M., Wang H.Z., Qiao Z.Y., Zhang J.T. (2023). Engineering of plasmonic metal-semiconductor yolk-shell nanostructures for multi-intensified photodynamic- and immuno- therapy against drug resistant bacteria. Nano Today.

[127] Overchuk M., Weersink R.A., Wilson B.C., Zheng G. (2023). Photodynamic and Photothermal Therapies: Synergy Opportunities for Nanomedicine. ACS Nano.

[128] Wang W., Wu F., Zhang Q., Zhou N., Zhang M., Zheng T., Li Y., Tang B.Z. (2022). Aggregation-Induced Emission Nanoparticles for Single Near-Infrared Light-Triggered Photodynamic and Photothermal Antibacterial Therapy. ACS Nano.

[129] Liu Y.-S., Wei X., Zhao X., Chen L.-J., Yan X.-P. (2021). Near-Infrared Photothermal/Photodynamic-in-One Agents Integrated with a Guanidinium-Based Covalent Organic Framework for Intelligent Targeted Imaging-Guided Precision Chemo/PTT/PDT Sterilization. ACS Appl. Mater. Interfaces.

[130] Li B., Wang W., Zhao L., Yan D.Y., Li X.X., Gao Q.X., Zheng J.D., Zhou S.T., Lai S.S., Feng Y. (2023). Multifunctional AIE Nanosphere-Based “Nanobomb” for Trimodal Imaging-Guided Photothermal/Photodynamic/Pharmacological Therapy of Drug-Resistant Bacterial Infections. ACS Nano.

